# Deep eutectic solvent self-assembled reverse nanomicelles for transdermal delivery of sparingly soluble drugs

**DOI:** 10.1186/s12951-024-02552-y

**Published:** 2024-05-21

**Authors:** Bin Li, Siwen Jiao, Shiqi Guo, Ting Xiao, Yao Zeng, Yingwei Hu, Xiaojuan Li, Sha Xiong, Yuehong Xu

**Affiliations:** School of Pharmaceutical Sciences, Sun Yat-sen University, Guangzhou, 510006 China

**Keywords:** Transdermal delivery, Deep eutectic solvent, Self-assembly, Reverse nanomicelle, Computational simulation, Anti-psoriasis

## Abstract

**Background:**

Transdermal delivery of sparingly soluble drugs is challenging due to their low solubility and poor permeability. Deep eutectic solvent (DES)/or ionic liquid (IL)-mediated nanocarriers are attracting increasing attention. However, most of them require the addition of auxiliary materials (such as surfactants or organic solvents) to maintain the stability of formulations, which may cause skin irritation and potential toxicity.

**Results:**

We fabricated an amphiphilic DES using natural oxymatrine and lauric acid and constructed a novel self-assembled reverse nanomicelle system (DES-RM) based on the features of this DES. Synthesized DESs showed the broad liquid window and significantly solubilized a series of sparingly soluble drugs, and quantitative structure-activity relationship (QSAR) models with good prediction ability were further built. The experimental and molecular dynamics simulation elucidated that the self-assembly of DES-RM was adjusted by noncovalent intermolecular forces. Choosing triamcinolone acetonide (TA) as a model drug, the skin penetration studies revealed that DES-RM significantly enhanced TA penetration and retention in comparison with their corresponding DES and oil. Furthermore, in vivo animal experiments demonstrated that TA@DES-RM exhibited good anti-psoriasis therapeutic efficacy as well as biocompatibility.

**Conclusions:**

The present study offers innovative insights into the optimal design of micellar nanodelivery system based on DES combining experiments and computational simulations and provides a promising strategy for developing efficient transdermal delivery systems for sparingly soluble drugs.

**Supplementary Information:**

The online version contains supplementary material available at 10.1186/s12951-024-02552-y.

## Background

Transdermal drug delivery system (TDDS) is a promising approach that delivers drugs into the skin tissue or through the skin for systemic action, which has advantages with avoidance of the liver first-pass effect, sustained and controlled delivery, and a non-invasive way [1]. However, the outermost stratum corneum (SC) of the skin is composed of corneocytes and lipid bilayers organized in a “brick and mortar”-like structure, comprising the main barriers against effective transdermal delivery [2]. Moreover, many commercially available active pharmaceutical ingredients present low solubility and poor permeability, especially those sparingly soluble drugs that are insoluble in neither water nor other conventional solvents, the development of their TDDS is a great challenge [3]. Some chemical penetration enhancers and solubilizers have been used for TDDS, but these ingredients probably exhibit skin irritation, allergic reactions, or other skin problems [4].

Recently, deep eutectic solvent (DES)-mediated TDDS has received increasing attention. DESs, recognized as ionic liquid (IL) analogs, are a class of green solvents formed from Lewis or Brønsted acids and bases, which exhibit ease of preparation, low vapor pressure, and high tunability, wherein DESs prepared from certain natural products exhibit good biocompatibility and biodegradability [5]. Numerous studies have shown that DESs can increase drug solubility, which is beneficial for the distribution and stability of drugs in formulations [6–8]. Additionally, DESs can improve skin permeability and promote drugs to better penetrate the skin barrier, thereby increasing transdermal absorption efficiency [9, 10]. Usually, the performance of DESs largely depends upon the types and proportions of used components. Mitragotri and coworkers developed a DES based on choline and geranate (CAGE) to transport macromolecular insulin transdermally [10]. The study showed that higher amounts of geranate variants in CAGE were more capable of transporting the drug owing to extracting more lipids than higher amounts of choline variants. In another study [11], the different amino acids were modified on the surface of mesoporous silica nanoparticles (MSNs) followed by formed DESs with citric acid, which could drive MSNs across into the deeper skin layers while DESs showed different delivery efficiencies depending on the amino acid type.

To achieve more effective drug delivery, ILs or DESs have been incorporated into other systems to design nanomedicine formulations. Compared to pure ILs/DESs, these delivery systems, combining the advantages of both nanocarriers and “green” solvents, provide exclusive drug delivery properties including reduced toxicity, enhanced penetration, selective targeting, and responsive release [12–15]. However, as far as we know, most of the DESs used are hydrophilic. Excessive dilution of DESs with water can destroy the hydrogen bonds between DES components, resulting in the loss of the supramolecular structure of DESs [16]. Moreover, additional auxiliary materials (surfactant/co-surfactant or organic solvent) are required to make them miscible with an oil phase when applied to oil-based formulations, which may cause skin irritation and potential toxicity after topical application repeatedly [17–19].

To overcome these limitations, we aimed to design an amphiphilic DES that can self-assemble into reverse nanomicelles (RM) by noncovalent intermolecular forces, i.e., DES-RM. Nanomicelles or RM are usually composed of self-assembled core-shell structures of amphiphilic molecules with a unique structure of having both hydrophilic and hydrophobic parts [20–22]. They are ideal for carrying bioactive molecules and hydrophobic drugs, and they will load a large amount of sparingly soluble drugs if amphiphilic molecules are replaced with DESs. Unlike the host-guest encapsulation through the porous and cavity structure of supramolecular carriers [23–26], drug loading in DES-RM may mainly depend on molecular miscibility. We previously screened DESs formed from the natural ingredient oxymatrine (OMT) and lauric acid (LA), which showed excellent solubilizing capacity on the sparingly soluble drug quercetin (QUE) and the proper hydrophilic-lipophilic balance characteristic [6]. These two unique attributes of OMT-LA DES will enable its use for RM preparation. As a novel nanocarrier for TDDS, DES-RM may have the following advantages. First, this DES is miscible with an oil phase without the necessity for additional auxiliary surfactants/co-surfactants to facilitate the formulation stability, which addresses the concerns of skin irritation and toxicity associated with chemical penetration enhancers. Second, DES-RM involves a core/shell nanostructure in which the shell composition (LA) has properties similar to the skin lipids. Hence, RM has good skin fusion, easily penetrates the skin barrier, and promotes the transdermal absorption of the drug. Third, RM may passively target the skin tissues through enhanced permeability and retention effects due to its nano-size structure, increasing the topical bioavailability of the drug [27, 28]. Therefore, it is hypothesized that the DES-RM possesses the advantages of solubilizing sparingly soluble drugs via DES and remarkably enhancing skin permeability via RM.

**Scheme 1:**
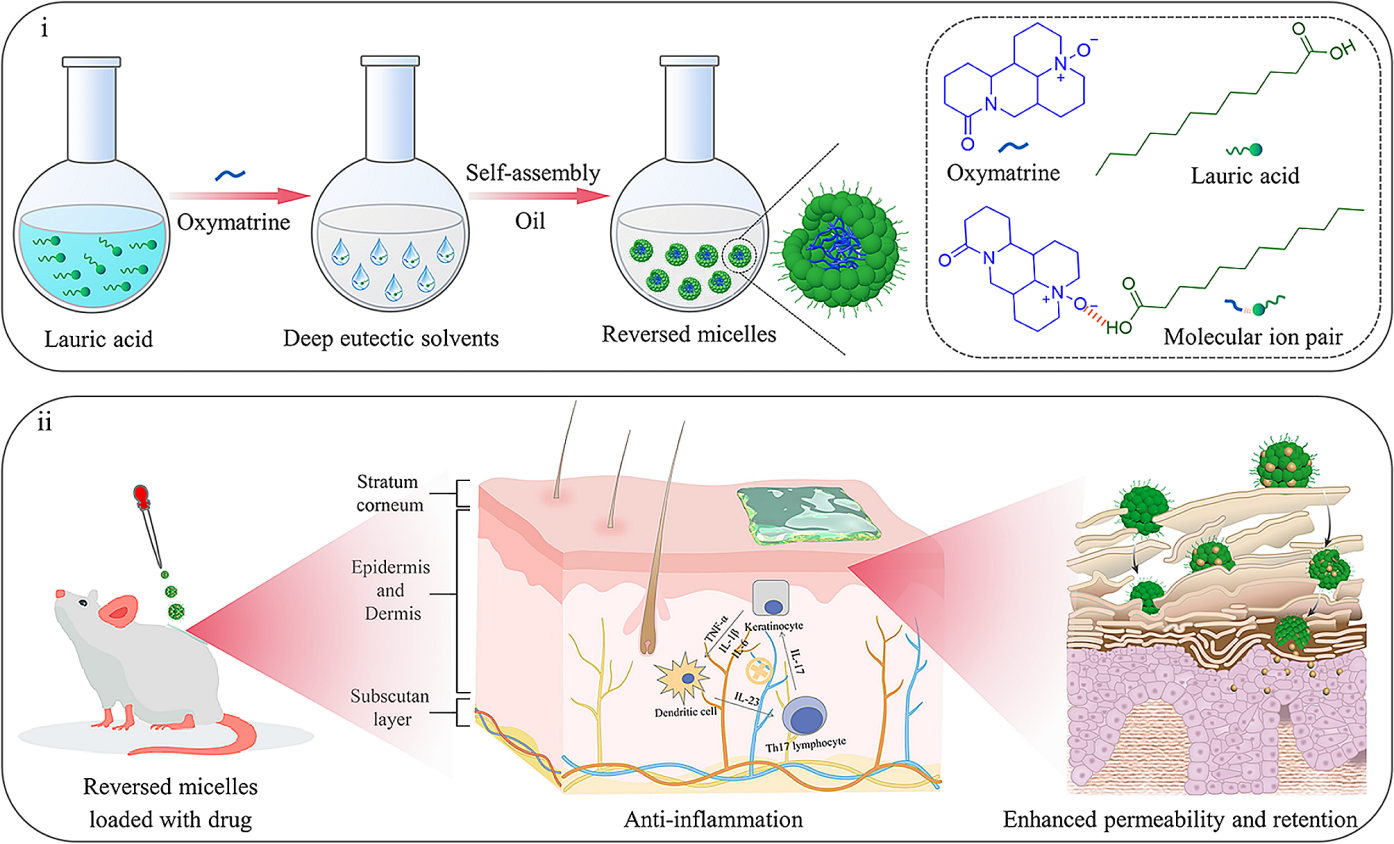
Schematic of DES-RM generation and transcutaneous treatment. (i) Charge-assisted hydrogen bonding can act as a ‘‘glue” to bond the head groups of OMT and LA together. These chain aggregates were further stacked through molecular van der Waals forces to form the core/shell nanostructure with an inner polar core and outer non-polar shell due to the solvophobic effect. (ii) DES-RM can deliver much TA into deeper dermal layers, thereby achieving excellent anti-psoriasis efficacy

Herein, DESs at different mole ratios of OMT/LA were firstly prepared and their structural and physicochemical properties were characterized by experimental and computational systematically. The solubilization effect of DESs on a series of sparingly soluble drugs was determined and analyzed using the quantitative structure-activity relationship (QSAR) models. Then, the formation mechanism of DES-RM was thoroughly revealed by combining spectral characterization and molecular dynamics simulations. Further, the skin permeability and possible mechanism of DES-RM were assessed. Finally, taking advantage of the combined effects of DES and loaded model drug triamcinolone acetonide (TA), an anti-psoriasis efficacy of the self-assembly system on the imiquimod (IMQ)-induced psoriasis mouse model was confirmed (Scheme 1). Taken together, this study suggests that DES-RM is a promising strategy for transdermal delivery of sparingly soluble drugs, and computation simulation is a companionship approach to comprehend the molecular mechanisms behind DES and DES-RM performance and to aid future formulation design.

## Materials and methods

### Materials

Minoxidil (MIN), piroxicam (PIR), baicalin (BAI), QUE, TA, OMT, and squalene were bought from Macklin (Shanghai, China). Adapalene (ADA) was purchased from Meiruier Chemical Technology Co., Ltd (Shanghai, China). LA and 8-hydroxypyrene-1,3,6-trisulfonic acid trisodium salt (HPTS) were purchased from Yuanye Biotechnology Co., Ltd (Shanghai, China). Ferulic acid (FA), castor oil, tea tree oil, and isopropyl myristate (IPM) were purchased from Aladdin (Shanghai, China). Acetonitrile and methanol were obtained from Sigma Aldrich (St. Louis, MO, USA). Dulbecco’s modified Eagle’s medium (DMEM) was purchased from Gibico (NY, USA). 3-(4,5)-dimethylthiahiazo (-z-y1)-3,5-di- phenytetrazoliumromide (MTT) was sourced from Solarbio (Beijing, China). Other reagents were obtained from commercial sources and used without further purification.

Sprague-Dawley (SD) rats and BALB/c mice were purchased from the animal center of Sun Yat-sen University (Guangzhou, China).

### Synthesis and characterization of DESs

DESs were fabricated using one-step method. In brief, OMT and LA were accurately weighed and dissolved in absolute ethanol at different molar ratios of 1:9, 2:8, 3:7, 4:6, 5:5, 6:4, 7:3, 8:2, and 9:1 at room temperature. The solvent was then removed by reduced-pressure rotary evaporation at 45 °C. Finally, the resulting mixtures were further dried in a vacuum oven for 48 h at 30 °C.

The theoretical eutectic point between OMT and LA was predicted with the Schroeder-van Laar equation [29]:

1 $${\text{In}}\;{{\text{x}}_i}{\gamma _i}\; = \; - \frac{{\Delta H}}{{RT}}\left( {1 - \frac{T}{{{T_m}}}} \right)$$

where *x*_i_ is the mole fraction, *γ*_i_ is the activity coefficient, Δ*H* is the melting enthalpy, *T*_m_ is the melting point, and *R* is the gas constant. Meanwhile, to simulate real conditions, a solid-liquid equilibrium phase diagram between OMT and LA was predicted by the Conductor-like Screening Model for Real Solvent (COSMO-RS) model in COSMOtherm software according to a method reported by Abranches et al. [30].

The melting point (*T*_m_)/glass transition temperature (*T*_g_) of DESs was measured using DSC 214 differential scanning calorimeter (NETZSCH, Germany) with a heating rate of 10 °C/min. Thermogravimetric analysis/derivative thermogravimetry (TGA/DTG) was performed on TG209F1 Libra thermogravimetric analyzer (NETZSCH, Germany) with a heating rate of 10 °C/min (N_2_ was used as a purge gas). Fourier transform infrared (FTIR) spectra were recorded between 4000 and 400 cm^− 1^ by Spectrum Two FTIR spectrometer (PerkinElmer Inc., USA) with a spectral resolution of 4 cm^− 1^. Proton nuclear magnetic resonance (^1^H NMR) spectra were recorded with an Avance III NMR spectrometer (Bruker, Germany). Nuclear overhauser effect spectroscopy (NOESY) experiment was carried out with an Ascend TM 500 NMR spectrometer (Bruker, Germany). Deuterated dimethyl sulfoxide (DMSO-*d*_6_) was used as a solvent with the solvent peak (*δ* 2.5, ppm) as a reference peak.

All-atom molecular dynamics (MD) simulations of DESs were carried out with the Materials Studio 8.0 software, the COMPASS force field was applied in all calculations. The quantum chemical calculations based on density functional theory (DFT) were also performed to clarify molecular interactions (Additional files 1 and 2).

### Property measurements of DESs

Viscosity was taken with controlled rate measurement from 0.1 to 100 s^− 1^ using a Kinexus pro + rotational rheometer (Malvern Instruments Ltd, UK). Before each test started, the sample was equilibrated for 5 min at 25 °C. The measurement was performed with a parallel plate geometry sensor (20 mm diameter) and a gap of 1 mm. The pH value was determined with an FE20 Plus pH meter (Mettler Toledo, USA). Before measurement, a two-point calibration was conducted with standard buffers. The conductivity measurement (µS/cm) of DESs with different water content (w/w) was taken using a DDSJ-318 conductivity meter (Shanghai INESA Scientific Instrument Co., Ltd., China). The microstructure was examined using an ECLIPSE LV100POL polarizing optical microscope (Nikon, Japan) to assess the stability of DESs during storage.

### Skin safety of DESs

Human immortalized epidermal cells (HaCaT) and human skin fibroblasts (HSF) were employed to evaluate the safety of DESs. Cells were cultured and grown until reaching the logarithmic phase in an atmosphere of 5% carbon dioxide at 37 °C. Subsequently, HaCaT and HSF cells were seeded at a density of 8000 cells per well on a 96-well plate and cultured for 24 h. Then, working solutions of DESs with different concentrations were added to the 96-well plate. After co-culturing for 24 h, the cell viability was measured by the MTT assay [6].

Histological analysis was further performed to evaluate the skin irritation of DESs. Briefly, hair from the back of the SD rat was first removed using an electric shaver. Then, DESs (0.3 mg) were evenly spread on the skin area of 2 × 2 cm^2^. After 24 h treatment, the treated skin area was cleaned, collected, and fixed with 4% paraformaldehyde, embedded in paraffin, sliced, and then stained with hematoxylin & eosin (H&E). Subsequently, sections were visualized under EVOS FL Auto Imaging System (Life Technologies, USA) at 10× magnification.

### Solubility study

The solubility of a series of sparely soluble drugs including MIN, PIR, TA, QUE, BAI, ADA, and FA was determined by the shake flask method. An excess amount of drugs was added into 2 mL of water, IPM, and DESs, respectively. All samples were then transferred to a constant temperature air bath oscillator and oscillated in the dark for 48 h at 32 °C. Undissolved drugs were centrifuged, and the supernatant was taken and diluted with methanol to the appropriate concentration. The drugs were determined using an LC-2030 Plus high performance liquid chromatography (HPLC) system equipped with a UV-Vis detector (Shimadzu, Japan). HPLC methods were provided in Table S1 (Additional file 3).

Additionally, QSAR models were applied to investigate the solubilization effect of DESs on various drugs (Additional file 3). The used molecular descriptors in this study included hydrogen bond donor (HBD) count, hydrogen bond acceptor (HBA) count, molar volume (*V*_m_), melting point (*T*_m_), topological polar surface area (*TPSA*), octanol-water partition coefficient (Xlog *P*), molecular refractivity (*MR*), solubility parameter (*SP*), and acidity coefficient (*pK*a).

### Self-assembly of DES-RM

Various pharmaceutically approved solvents (such as fatty acid esters: IPM and castor oil, plant essential oils: tea tree oil, and terpenes: squalene) were used to evaluate the self-assembly ability of DESs in the hydrophobic environment. In brief, 0.25 g of DESs and 0.25 g of the above solvents were stirred in a clear glass bottle at room temperature for 3 min. Whether a clear liquid without precipitation was visually formed indicated the intersolubilizaiton of DESs with the oil phase to be self-assembly. The self-assembly of DESs in an oil phase IPM was investigated using a Zetasizer Nano-ZS Particle Sizer (Malvern Instruments Ltd., UK) by dynamic light scattering (DLS) technology. The critical reverse micelle concentration (CRMC) of DESs was detected using a pyrene radiometric method. Briefly, the pyrene was dissolved in methanol overnight, with a concentration of 1 mM. Then, 10 µL of pyrene solution was added into 990 µL of IPM with various concentrations of DESs. After incubating at 37 °C for 4 h in the dark, the fluorescence intensity was detected by a Synergy H1 microplate reader (BioTek, USA) with an excitation wavelength of 335 nm. The fluorescence intensity ratio values of I_392_/I_375_ were recorded for CRMC evaluation [18]. The TEM image of the self-assembly was taken at 200 kV using a JEM-2010 transmission electron microscopy (JEOL, Tokyo, Japan). The sample solution of 2.5 µL was placed on a carbon-coated copper grid for 2 min, rinsed three times with cyclohexane, and stained with 2% phosphotungstic acid for 3 min [31]. The copper mesh slice was further dried overnight in a vacuum oven at 30 °C. FTIR and conductivity were performed as the aforementioned.

MD simulation was performed to directly observe the self-assembly process at the molecular level. The low-concentration (10%, w/w) and high-concentration (50%, w/w) systems of DESs composed of twenty molecules and fifty molecules in an IPM box sized 4 × 4 × 4 nm were constructed, respectively. Among them, the number ratios of OMT/LA were set as 5/5, 4/6, and 3/7, respectively. After initial geometry optimization, constant temperature, constant volume (NVT) and constant temperature, constant pressure (NPT) simulations of 2.0 ns were performed, respectively.

### Skin penetration studies

Based on the results of the solubility study, TA was chosen as a model drug to evaluate the effect of DES-RM on drug permeability. According to our previously reported method [6], rat skin was carefully placed on the Franz diffusion cell (3.14 cm^2^). Then, 0.3 mg of DES (4:6)-RMs with different DES concentrations (the DES concentration was 0%, 10%, 20%, 30%, 40%, 50%, 100%, respectively; and TA concentration was 0.1% in each sample, w/w) were added to the donor cell, covering the surface of SC. The receptor cell was filled with 8 mL of PEG 400-normal saline (v/v, 30:70) at 32 °C. The receptor medium (1 mL) was collected at the predetermined time points and simultaneously an equal amount of fresh medium was compensated to the receptor cell to keep the solution volume constant. After the permeation test, the skin was rinsed and chopped into small pieces, then extracted with 1 mL of methanol to determine the topical delivery content. The TA accumulated in the receptor medium and skin was determined by HPLC. The cumulative penetration amount (*Q*) was calculated as:

2 $$Q\; = \;\frac{{8\left( {{C_n}\; + \;\sum\limits_{i = 0}^{i = n - 1} {{C_i}} } \right)}}{{3.14}}$$

where *C*_n_ is the drug concentration in the receptor medium at time point, *t*.

Additionally, the permeation-enhancing effect of DES-RM on the drug across the skin was visualized using confocal laser scanning microscopy (CLSM). DES-RM at different mole ratios of OMT/LA (5:5, 4:6, and 3:7) was prepared to contain coumarin-6 (C6, a fluorescent agent) of 0.1% (w/w) and performed in vitro penetration test for 4 h. After that, the skin samples were removed and cleaned with normal saline. An FV3000 laser scanning microscope (OLYMPUS, Japan) was performed to directly examine the fluorescent intensity of C6 in the different skin layers at 10× magnification. The skin surface was set as the imaging plane (z = 0 μm) with a scanning interval of 10 μm.

### Mechanism of penetration enhancement

The skin treated with DES-RM was observed using a scanning electron microscope (SEM) and FTIR to clarify the penetration-enhancing mechanism. As described in the aforementioned, the skin was permeated with DES-RM at different mole ratios of OMT/LA (5:5, 4:6, and 3:7) for 4 h. After wiping with normal saline, skin samples were fixed with 4% paraformaldehyde overnight and dehydrated in gradient ethanol solutions at 30%, 50%, 70%, 90%, and 100% (v/v) [11]. Afterward, the skin samples were further subjected to critical point drying and gold sputtering and visualized by a JSM-6330F cold-field emission scanning electron microscope (JEOL Ltd., Japan).

The structural changes of SC were quantitatively detected by FTIR spectroscopy [10]. Before the experiment, the fresh skin was dried for 8 h in the fume hood. A control FTIR spectrum was recorded based on the accumulation of 8 scans in a wavenumber range of 4000 –400 cm^− 1^ on a Spectrum Two FTIR spectrometer (PerkinElmer Inc., USA) equipped with an attenuated total reflection (ATR) device. Then, the skin was permeated with DES-RM at different mole ratios of OMT/LA (5:5, 4:6, and 3:7) for 24 h, skin sheets at the application site were collected and further dried for 24 h at room temperature. A second FTIR spectrum was taken and compared with the control spectrum to evaluate the effect of DES-RM on the SC structure. The integrated area (*AUC*) of specified peaks was calculated using Origin 2018 software (OriginLab Corporation, USA).

### In vivo therapeutic efficacy

The IMQ-induced psoriatic mouse model was established to evaluate the therapeutic efficacy of the formulation [32]. Based on the above results, 10% DES (4:6)-RM was selected as the topical delivery carrier of TA, namely TA@DES-RM (containing 0.1% TA, w/w). The BALB/c mice (male, 6–8 weeks old) were randomly divided into five groups: (1) control group; (2) model group; (3) blank DES-RM group; (4) TA@DES-RM group; and (5) commercial TA cream (0.1%, w/w, FRONT PHARMACEUTICAL, Anhui, China). The mice were raised for one week for adaptation before the experiment (*n* = 7 per group). Subsequently, mice were depilated on their backs and restored for 24 h. Except for the control group, the other four groups of mice were given 5% IMQ cream (Aldara, Ensign Laboratories Pty Ltd, Singapore) every day (62.5 mg/day). After 4 h, TA cream, DES-RM, and TA@DES-RM of 50 mg were administered for treatment respectively.

During the treatment period, body weight and psoriasis area and severity index (PASI) score of mice were recorded to assess the severity of psoriasis every day. The dorsal skin and spleen of mice were collected after mice were sacrificed on day 7. The spleen index was calculated as spleen weight (mg)/mice weight (g). The skin samples were fixed in 4.0% paraformaldehyde and embedded in paraffin for H&E and immunohistochemical staining. The slices were observed under an EVOS M7000 Imaging System (Thermo Fisher Invitrogen, USA). In addition, the mRNA expression levels of inflammatory factors including tumor necrosis factor-α (TNF-α), interleukin-1β (IL-1β), IL-6, IL-17 A, and IL-23 in the skin samples were also determined with quantitative real-time polymerase chain reaction (qRT-PCR) [33]. The sequences of specific primers used are shown in Table 1.

**Table 1:** Sequences of the primers used for qRT-PCR analysis

Gene	Forward (5′ to 3′)	Reverse (5′ to 3′)
GAPDH	TAGCCCACGTCGTAGCAAAC	TACGGCCAAATCCGTTCACA
TNF-α	TAGCCCACGTCGTAGCAAAC	ACCCTGAGCCATAATCCCCT
IL−1β	TGCCACCTTTTGACAGTGATG	TTCTTGTGACCCTGAGCGAC
IL−6	GGGACTGATGCTGGTGACAA	CGCACTAGGTTTGCCGAGTA
IL−17 A	GCTGACCCCTAAGAAAACCCC	GAAGCAGTTTGGGACCCCTT
IL−23	CAAAGGATCCGCCAAGGTCT	CTTGCCCTTCACGCAAAACA

### Statistical analysis

The results were expressed as mean ± standard deviation (SD) of at least three independent experiments. Differences were evaluated using a two-sample t-test or analysis of variance (ANOVA) with post-hoc Tukey’s tests (Origin 2018). Value with *P* < 0.05 was considered statistically significant.

## Results and discussion

### Preparation and characterization of DESs

**Fig. 1:**
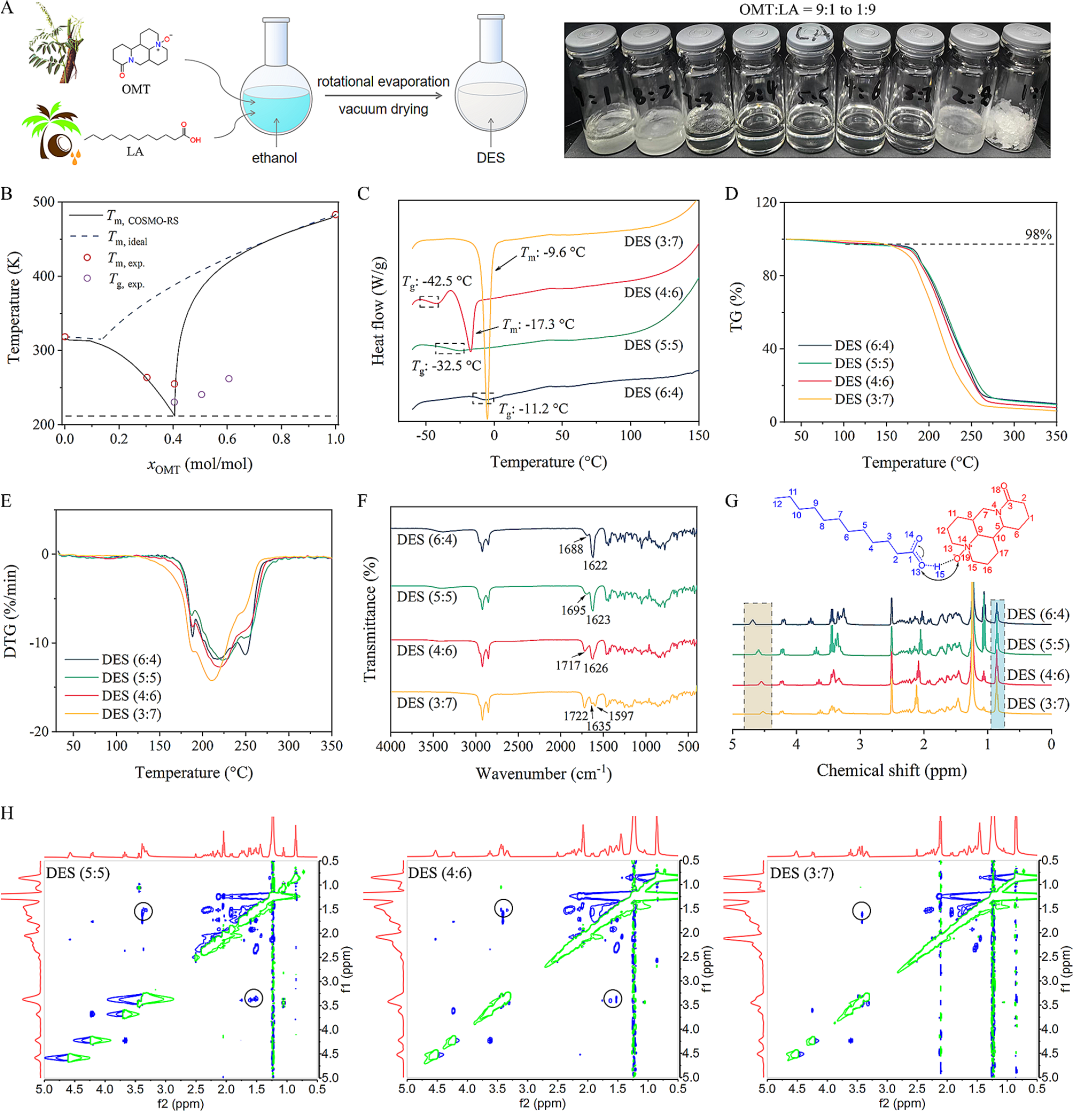
Preparation and characterization of DESs. (**A**) Preparation process and appearance photos of DESs; (**B**) Solid-liquid phase diagram of OMT + LA binary mixtures; (**C**) Differential scanning calorimetry (DSC) thermograms of DESs; (**D**) Thermogravimetry analysis (TGA) of DESs; (**E**) Differential thermogravimetry (DTG) curves of DESs; (**F**) Fourier transform infrared (FTIR) spectra of DESs; (**G**) Proton nuclear magnetic resonance (^1^H NMR) spectra (400 MHz, DMSO-*d*_6_) of DESs. (H) 2D Nuclear overhauser effect spectroscopy (NOESY) spectra (500 MHz, DMSO-*d*_6_) of DESs. The cross-peaks (interactions) between H_13_ and H_15_ of OMT and H_3_ of LA were circled in black

DESs are usually composed of at least one HBD and one HBA, which presents a deeper depression in the melting point when combined at a suitable stoichiometric ratio. Here we synthesized DESs using OMT and LA by one-step method (Fig. 1A). To distinguish whether the blend is a simple eutectic mixture or a “deep” eutectic solvent, we first established the solid-liquid phase diagram of the binary system. Assuming that OMT and LA are miscible, the activity coefficient approaches unity, so eutectic behavior can be represented as an ideal V-shaped phase diagram [29]. While deep eutectic is exhibiting non-ideal negative deviation. As shown in Fig. 1B, the ideal eutectic point of OMT and LA was about 41 °C with a 0.14 mol fraction of OMT. In terms of appearance, blends with OMT/LA mole ratios of 9:1, 8:2, 7:3, 2:8, and 1:9 were presumed to be partially melted and presented as a mixture of solid and liquid or wet pasty (Fig. 1A). At OMT/LA mole ratios of 6:4, 5:5, 4:6, and 3:7, blends appeared to be in a homogeneous liquid state at room temperature, which implied that such liquid mixtures belong to DES.

DSC thermograms showed that liquid mixtures at these mole ratios began to melt at lower temperatures compared with OMT or LA alone and appeared very significant negative deviation from ideality (Fig. 1B and C; additional file 4). The COSMO-RS model reasonably described the non-ideality of the OMT/LA system, and the eutectic point occurred at the OMT/LA mole ratio of 4:6. Thus, the formation of DESs at wide mole ratios was further confirmed by comparing an experimental curve with an ideal curve, and they were named DES (6:4), DES (5:5), DES (4:6), and DES (3:7) in the following investigation. It is worth noting that DES (6:4), DES (5:5), and DES (4:6) exhibited an evident *T*_g_, suggesting their non-crystalline nature. Among them, DES (4:6) accrued a recrystallization event followed by corresponding to the melting of the crystalline mixture. However, DES (3:7) only showed a single sharp peak with a melting temperature of -9.6 °C. These results suggested that DES aggregation changed gradually from amorphous to crystalline with the increase of the mole fraction of LA.

To estimate the thermal stability of the synthesized DESs, TGA/DTG thermograms were recorded from 30 to 350 °C (Fig. 1D and E). It can be seen that the mass losses of DESs mainly occurred at two stages. The initial mass losses (0.8–1.5%) occurred in the temperature ranges of 50–120 °C, which may be attributed to the evaporation of adsorbed water molecules [12]. The mass losses (89–93%) of the second stage started at near 170 °C, this corresponded to the actual thermal decomposition of DESs. Moreover, the decomposition temperatures of DESs were between OMT and LA (Additional file 4), indicating that DESs had good thermal stability. Peak temperatures for DTG curves showed the maximum rate of mass loss in the temperature ranges of 187–250 °C. The thermal stability of DESs at different mole ratios mainly depended on the composition fraction of LA, because LA was relatively easier to decompose at a high temperature.

Furthermore, DESs were characterized by FTIR, ^1^H NMR, and 2D NOESY which provided information confirming the intermolecular interactions of DESs. The peaks at 1606 and 2287 cm^− 1^ were attributed to the C = O vibration and the N^+^-O^−^ vibration of OMT (Additional file 4). After forming DESs, the N^+^-O^−^ vibration of OMT disappeared. Meanwhile, a blue shift in C = O stretching vibration was also observed and increased with decreasing proportions of OMT (DES (6:4), 1622 cm^− 1^; DES (5:5), 1623 cm^− 1^; DES (4:6), 1626 cm^− 1^; DES (3:7), 1635 cm^− 1^). This may be related to the changes in the molecular environment and hydrogen bonding partner. On the other hand, when LA was present alone, the peak at 1697 cm^− 1^ for the carboxyl group was very sharp; however, with the addition of OMT, the intensity of the peak weakened (Fig. 1F), possibly due to the involvement of OMT in new intermolecular interaction formation. According to our previous report [6], this was confirmed to be due to the presence of charge-assisted hydrogen bonding. In addition, the vibration frequency of LA carboxyl group increased as the proportion of LA increased, confirming the dissociation of carboxylic acid dimeric structure and growth of one hydrogen bond pattern [34].

^1^H NMR spectra of DESs in DMSO-*d*_6_ were shown in Fig. 1G. The peak areas OMT in *δ* = 5.01 ppm (H_5_) and LA in *δ* = 0.85 ppm (H_12_) were chosen as the benchmark, respectively. We could observe that the values of *δ*_5.01_/*δ*_0.85_ were approximately equal to 1.32, 1.06, 0.65, and 0.42 for DES (6:4), DES (5:5), DES (4:6), and DES (3:7), which corresponds to the molar ratios of OMT/LA of 6:4, 5:5, 4:6, and 3:7, respectively. Thus, the successful synthesis of DESs containing OMT and LA in a stoichiometric ratio was further confirmed by combining the DSC and ^1^H NMR analysis. Also, the part proton signals of OMT inside DESs presented obvious changes, with further upfield or downfield chemical displacement. These protons were concentrated on the 5th, 13th, and 15th carbon atoms (*δ* = 5.02, 3.22, and 2.82 ppm), presumably due to the effect of the electrostatic interaction with LA carboxyl group [35]. The disappearance of the proton signal at the carboxyl group of LA in all DES spectra comparison. While in the spectrum of LA alone, the proton signal was observed in the *δ* = 11.94 ppm. These results indicated that hydrogen bonding may be established between the N-oxide from OMT and the carboxyl group from LA. In addition, NOESY was used to investigate the intermolecular interactions occurring in DESs. The cross-peaks reveal the spatial correlation between the protons. As indicated in Fig. 1H, multiple H-H cross-peaks were identified in the NOESY spectra of DESs, indicating the presence of multiple NOE between the two components. This supramolecular network was the characteristic of DES. In the case of DES (5:5) and DES (4:6), obvious interactions were observed between the protons (H_13_ and H_15_) surrounding the nitroso of OMT and protons (H_3_) surrounding the carboxyl group of LA. For DES (3:7), this correlation weakened and interactions between the protons (H_2_) surrounding the carboxyl group of LA increased, which was attributed to the molar excess of LA. Thus, these DESs had different microscopic interactions on a molecular level and might have different properties.

**Fig. 2:**
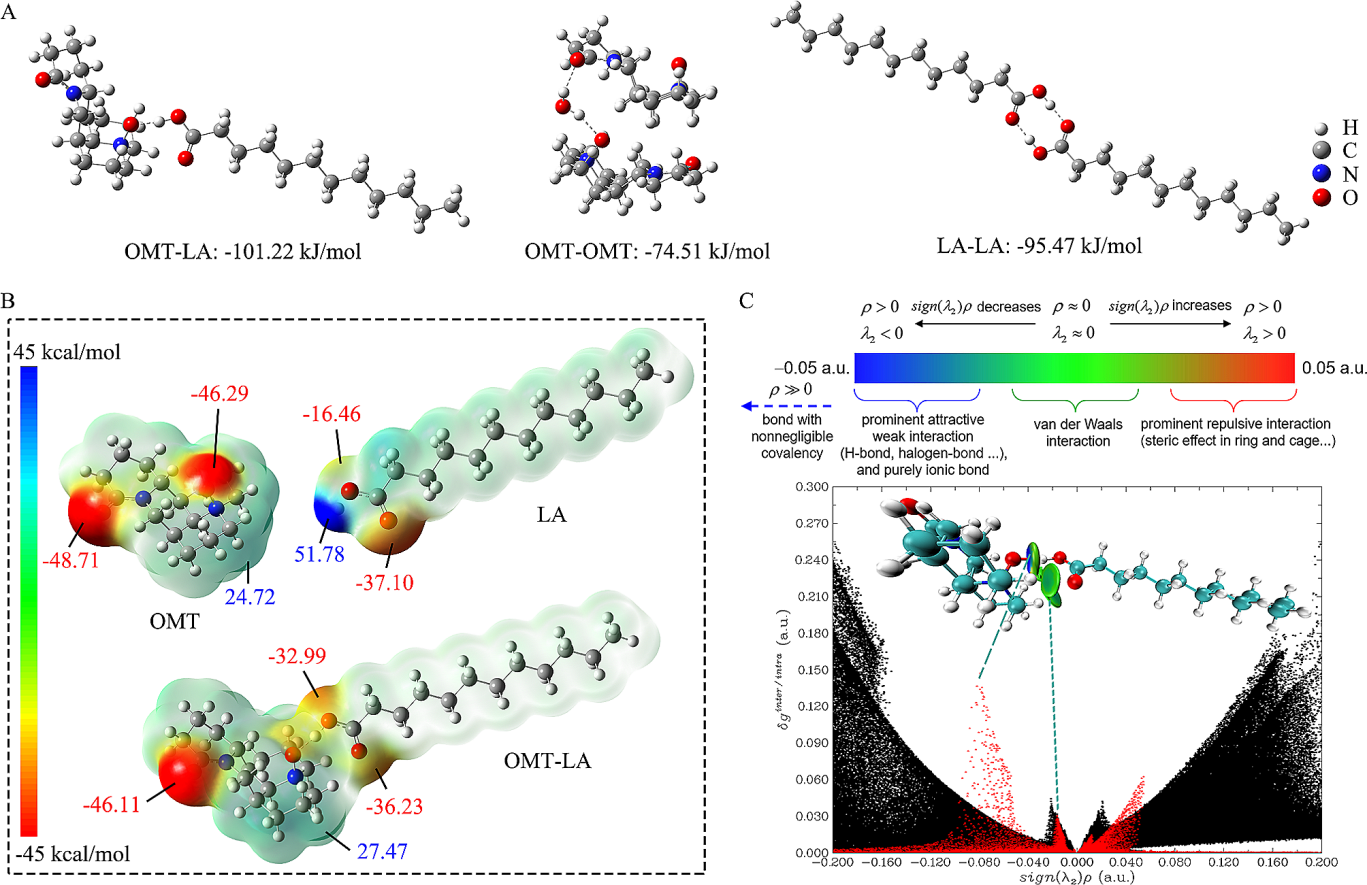
Density functional theory (DFT) calculations of DESs. (**A**) Geometries and interaction energies of most probable pairs of interacting molecules in DESs, pure OMT, and pure LA; (**B**) Molecular electrostatic potential (ESP) on the van der Waals surface (0.001 a.u.) of OMT, LA, and OMT-LA molecular ion pair; (**C**) Scatter plots between *δ*g vs. Sign (*λ*_2_) *ρ* of OMT-LA molecular ion pair and corresponding color-filled isosurface through the independent gradient model based on Hirshfeld partition (IGMH) analysis

MD simulations (Additional file 1) further presented the supramolecular network formed by hydrogen bonds at different mole ratios of DESs (e.g. O-H (LA)•••N^+^-O^−^ (OMT), O-H (LA)•••C = O (OMT)). Nevertheless, there was the strongest correlation between the O_19_ atom at the N-oxide of OMT and the H_15_ atom at the carboxyl group of LA for all DESs, which means that the heterodimer synthons consisting of O-H (LA)•••N^+^-O^−^ (OMT) hydrogen bonds play a dominant role in the DES formation. DFT calculations were further applied to confirm the formation mechanism of DESs. The strongest pair-wise interaction for each possible combination (OMT-OMT, LA-LA, and OMT-LA) was considered in the calculations. The results from interaction energy (Δ*E*) suggested that the hydrogen bonding established between OMT and LA was much stronger than the hydrogen bonding when pure substances were present alone (Fig. 2A). This might be due to the oxygen binds with the nitrogen atom through the coordination bond, and even though reducing the alkalinity of OMT, the lone electron of the oxygen makes it more polarity. As such, N-oxide became a better hydrogen bond acceptor than a carbonyl group. In fact, OMT exists in the hydrate form under natural conditions [36]. When OMT and LA are mixed, OMT preferentially forms hydrogen bonds with LA since the hydroxyl group of LA is a stronger HBD than that of water. The above assumption was further supported by electron density analysis (Additional file 2). The color depth in the molecular surface illustrated the intensity of electrostatic potential (ESP). As shown in Fig. 2B, the negative ESP regions of OMT were mainly located on oxygen atoms, while the positive ESP region of LA was mainly located on the carboxyl hydrogen atom. For the dimer formed by OMT and LA, the positive region of LA was attracted to the negative region of OMT, thereby leading to electron transfer and delocalization [37]. According to the independent gradient model based on Hirshfeld partition (IGMH) analysis, non-covalent interactions between OMT and LA were visualized (Fig. 2C). IGMH can intuitively display the interaction intensity and characteristics between different fragments [38]. It could be seen that the strong electrostatic interaction and van der Waals attraction between OMT and LA played an important role in the binding of the two species.

### Basic properties of DESs

**Fig. 3:**
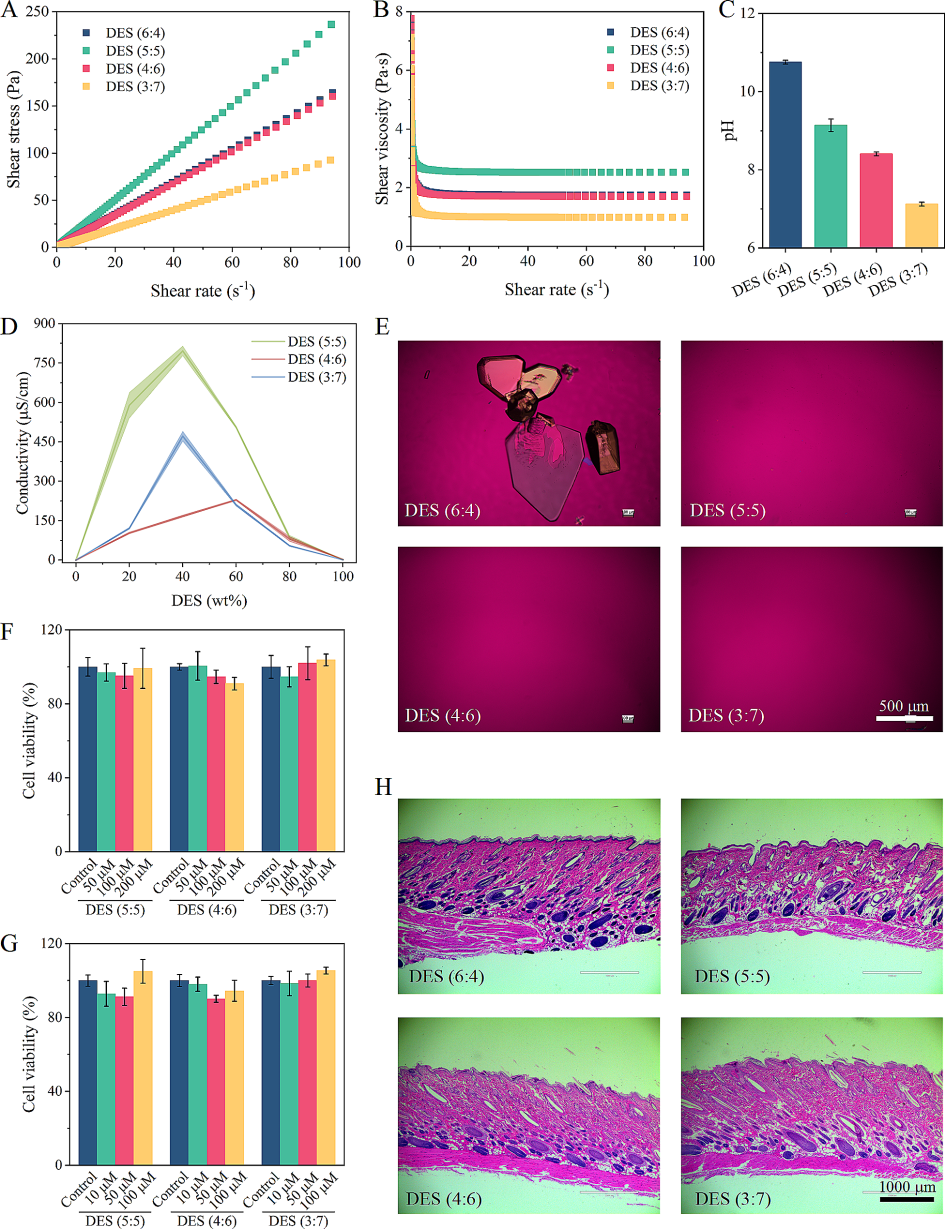
Physicochemical properties and skin safety of DESs. (**A**) Shear stress-shear rate relationship of DESs; (**B**) Shear viscosity-shear rate relationship of DESs; (**C**) The pH values of DESs; (**D**) Conductivity of DESs under different water contents; (**E**) Stability of DESs under storage at room temperature; (**F**) The viability of Human immortalized epidermal cells (HaCaT) after incubation with DESs; (**G)** The viability of human skin fibroblasts (HSF) after incubation with DESs; (**H**) Histopathological examination of rat skin treated with DESs for 24 h

The physicochemical properties of DESs are crucial to their development and application of transdermal formulations. We determined the properties of DESs including viscosity, pH, conductivity, and stability. From the flow curves at a shear rate from 0.1 to 100 s^− 1^, all synthesized DESs suggested Newtonian behavior (Fig. 3A). The mole ratios of OMT/LA significantly influenced the viscosity of liquid mixtures, and the viscosity was between 1.0 and 2.5 Pa∙s (Fig. 3B). The prepared DESs showed pH values ranging from 7.1 to 10.8 at room temperature (Fig. 3C). With the increase of LA mole ratio, the pH of DESs gradually decreased. This is due to that pH reflects the scale of acidity of a liquid depending on the relative acidity or basicity of materials that are mixed, as well as their stoichiometry [39]. Considering that the water content in the skin increases up to 60% as the depth increases [40], we also evaluated the effect of water addition on the supramolecular structure of DESs by conductivity measurement. Generally, excessive dilution of DES can destroy hydrogen bond interactions between components, allowing charges to migrate more readily through the solution, thus resulting in increased conductivity [16]. Figure 3D shows the conductivity results under different water content. The conductivity substantially increased and then decreased with the increase of the water content, displaying the highest value at the range of 40–60 wt% water. In addition, when adding water into the DESs, we observed the formation of a gel-like substance in DES (4:6) (Additional file 4), which may be attributed to hydrophobic interactions between the tails of LA and LA anion [41]. The increase in viscosity caused a lower conductivity of DES (4:6) + water mixtures, given the diffusion of molecules [42]. That is, the cluster structures of DESs were probably destroyed gradually when the amount of water was high, owing to the hydration. Additionally, recrystallization was observed for DES (6:4) during storage (less than one month). Polarized light microscopy appearing birefringence also confirmed the presence of crystalline substances (Fig. 3E). This behavior might be attributed to the crystal growth of an excess of OMT by absorbing water. In contrast, DES (5:5), DES (4:6), and DES (3:7) were still stable enough to maintain homogeneous liquid for at least one year.

Skin toxicity/irritation of DESs was assessed using two cell models and histopathological examination of skin. The poor stability of DES (6:4) limited its application, only DES (5:5), DES (4:6), and DES (3:7) were selected for further analysis. As shown in Fig. 3F and G, DESs at different mole ratios had no cytotoxicity on HaCaT and HSF at tested concentrations, showing a wide safety range. Furthermore, histopathological examination showed that no obvious loose edema, necrosis, and inflammatory cell infiltration were observed for DES groups in comparison with the control skin (Fig. 3H), which implied good biocompatibility. From these results, DESs formed from OMT and LA with suitable physicochemical properties and skin safety are worth further exploring for application in transdermal delivery systems.

### QSAR analysis of drug solubilization

**Fig. 4:**
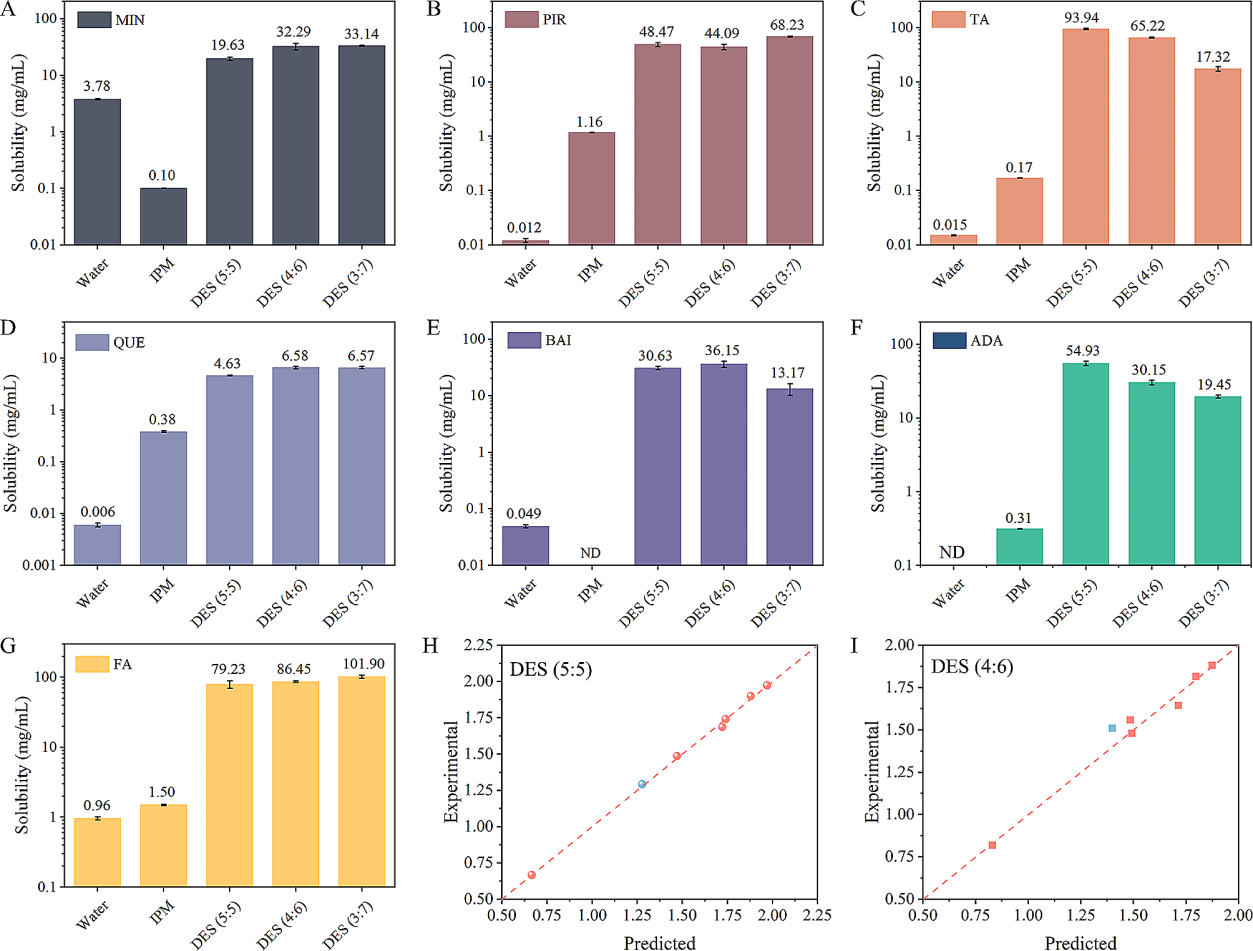
Solubility of various drugs and quantitative structure-activity relationship (QSAR) analysis. (**A**) Minoxidil (MIN); (**B**) Piroxicam (PIR); (**C**) Triamcinolone acetonide (TA); (**D**) Quercetin (QUE); (**E**) Baicalin (BAI); (**F**) Adapalene (ADA); (**G**) Ferulic acid (FA); (**H** and **I**) Plots of experimental versus predicted solubility based on the genetic function approximation (GFA) model

Formulation development of sparingly soluble drugs is very challenging. Hence, the solubilization ability of DESs on different drugs was measured and compared with that in water and a commonly used hydrophobic oil solvent IPM. Figure 4A-G presents the solubility results of these drugs including MIN, PIR, TA, QUE, BAI, ADA, and FA. It can be seen that MIN and BAI were more soluble in water than in the oil, while PIR, TA, QUE, ADA, and FA were more soluble in the oil. However, the solubility of these drugs was very low in both the water and oil. In contrast, the solubility of all drugs in the DESs was significantly enhanced. Among them, TA solubility in the DES (4:6) increased by 4348-fold compared with that in the water and increased by 384-fold compared with that in the oil.

The solubilization mechanism of DESs on various drugs was further studied by QSAR. In additional file 3, the input data containing drug structural and topological descriptors were shown, as well as the dependent variable logarithm of solubility (log *S*). The training (6 drugs) and test (1 drug) set were randomly selected. The independent variables were first selected using correlation analysis. Descriptors with high cross-correlation values (> 0.7) were not considered. Genetic function approximation (GFA) and partial least square (PLS) were then used to carry out the linear regression analysis. The results showed that GFA models containing *T*_m_, Δ*SP*, and *pK*a could well describe the changes in log *S*. The adjusted R^2^ was similar to the goodness of the model R^2^, indicating that there was no overfitting. The predicted value of the test drug (MIN) compared with the experimental value was within the applicability domain (Fig. 4H and I). The linear regression equations for DESs were listed as follows:

3 $$\eqalign{{\text{DES }}\left( {5:5} \right)\;:\;\log S &= - 0.1890{T_m} - 0.4508\Delta SP \cr &\quad+ 0.1161pKa + 1.5138\,{R^2} = 0.9983,{R^2}_{adj} \cr &\quad= 0.9957,{Q^2} = 0.9916,s \cr &\quad= 0.0155,F = 390.45,p < 0.05}$$

4 $$\eqalign{{\text{DES }}\left( {4:6} \right):\;\;\log \;S\; &= \; - 0.2279{T_m}\; - \;0.2916\Delta SP\; + \;0.1222pKa\; \cr &\quad+ \;1.4642{R^2} = 0.9850,{R^2}_{adj} \cr &\quad= 0.9625,{Q^2} = 0.5743,s\cr &\quad = 0.0368,F = 43.72,p < 0.05 }$$

Regrettably, the solubilizing behavior of DES (3:7) on drugs could not be well predicted (*p* > 0.05), which might be attributed to the influence of more factors. *T*_m_ reflects the crystal lattice energy of molecules. It has been previously shown that for drugs with a *T*_m_ of more than 200 °C, the crystal lattice has a strong influence on the solubility [43]. *SP* reflects the interaction energy between molecules. According to the solubility parameter theory introduced by Hildebrand and Scott, mutual solubility would be higher if the *SP* values of the drug/solvent are closer [44]. *pK*a reflects the ability to accept electrons or donate electrons, mainly affecting the ionization degree of the drug [45]. Based on the solubility results of different drugs, good molecular compatibility could more efficiently solubilize drugs (e.g. ADA and TA), while the presence of acid groups had little effect on solubility. This was consistent with the results of correlation analysis that there was the strongest relation between log *S* and Δ*SP*, followed by *T*_m_ and *pK*a (Additional file 3). In general, our prepared DESs showed good solubilization performance on different drugs, and the established model could be extrapolated and used to predict the solubility of other transdermal drugs.

### Self-assembly mechanism of DESs

**Fig. 5:**
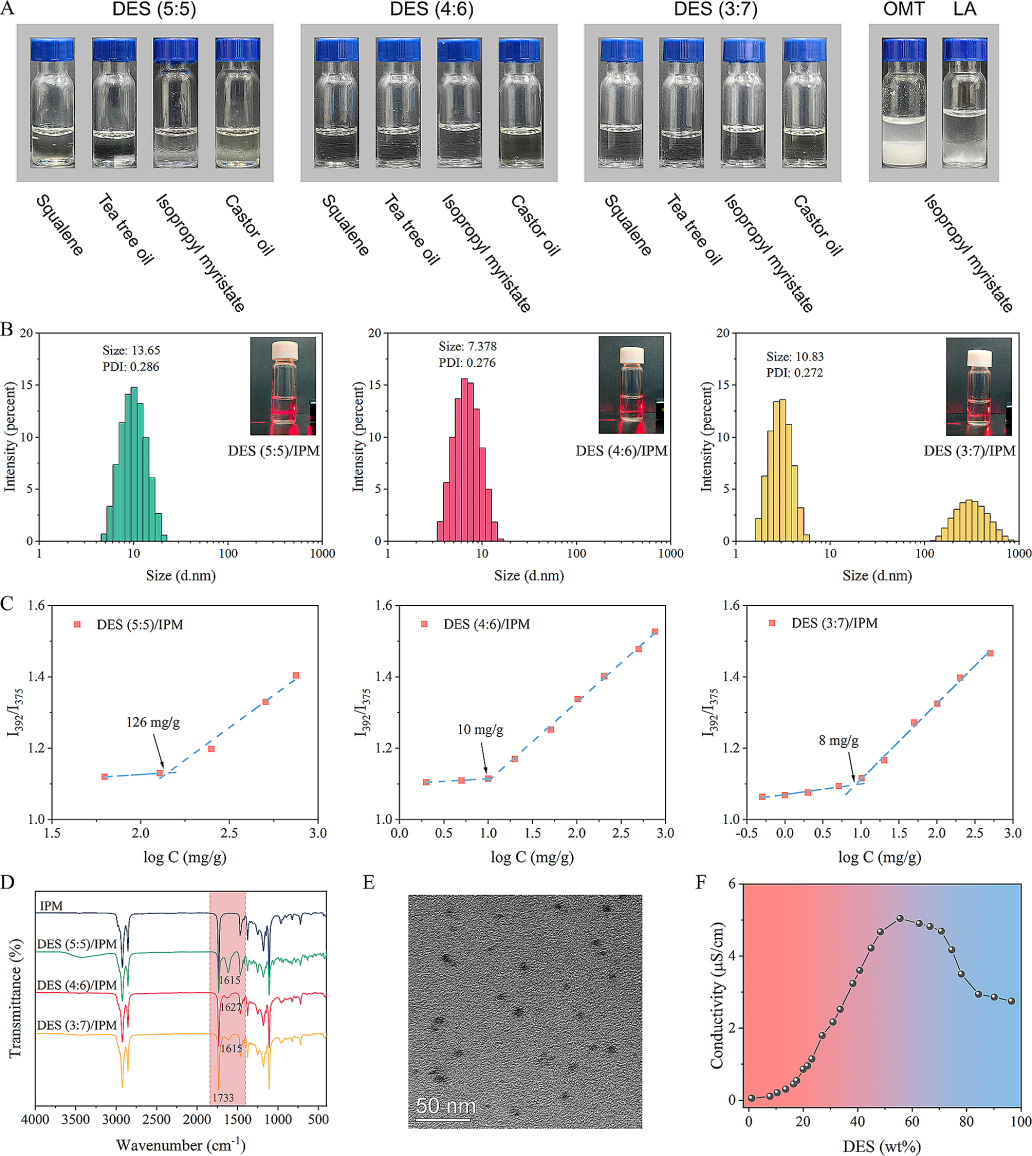
Self-assembly mechanism of DESs. (**A**) Intersolubility of DESs with pharmaceutically acceptable oil phases; (**B**) Size distribution of the reverse micelles (20% DES-RM); (**C**) The critical reverse micelle concentration (CRMC) detection of DESs by pyrene radiometric method; (**D**) FTIR spectra of 20% DESs dispersed in IPM; (**E**) Transmission electron microscope (TEM) imaging of 20% DES (4:6)-RM; (**F**) Plot of conductivity as a function of DES (4:6) mass fraction in IPM. The curve was divided into three different subdomains: DES-in-oil (< 45%), bicontinuous (45–59%), and oil-in-DES (> 59%)

To clarify the self-assembly behavior of synthesized DESs in non-polar media, we preliminary assessed their intersolubility with various oil phases. Figure 5A shows that DES (5:5), DES (4:6), and DES (3:7) can be dispersed evenly in IPM, castor oil, tea tree oil, and squalene to form an optically transparent solution, especially for DES (4:6) and DES (3:7) which can be dispersed in the oil at any ratio. In contrast, pure OMT created a granular precipitate, while the recrystallization occurred quickly (< 15 min) for LA dissolved in the oil phase. We inferred that DESs composed of hydrophilic OMT and hydrophobic LA probably formed a nanostructure with RM characteristics through the solvent-induced self-assembly effect [46, 47]. Further investigation elucidated the self-assembly mechanisms as follows.

DLS was used to measure the particle size and distribution of the self-assembly. The RM-forming tendency of DESs was confirmed by measuring the CRMC using pyrene as a fluorescent probe. The micromorphology of RM was characterized by TEM. FTIR and conductivity measurements were used to explore the interactions and structural features inside DESs. Figure 5B shows the size and distribution of the self-assembly. Three DESs dispersed in the oil phase exhibited nano-size with 13.65, 7.38, and 10.83 nm, respectively. A clear Tyndall light-scattering effect was observed. The CRMC of DES (5:5), DES (4:6), and DES (3:7) was 126, 10, and 8 mg/g, respectively, verifying the existence of a nano-structure of RM (Fig. 5C). Figure 5D and additional file 5 display the FTIR spectra of DESs dispersed in IPM. Compared with characteristic vibration frequencies of components of DESs, no obvious change was observed, which indicated that basic structural units of DESs still were retained in the oil phase. Moreover, the spectra of DESs dispersed in IPM were also compared with the spectra of DESs dispersed in water. With increasing water content, the carbonyl vibration of OMT gradually red-shifted and the carboxyl vibration of LA disappeared (Additional file 5), confirming the dissociation and hydration of DESs. A previous study has shown a connection between hydrogen bond strength and microenvironment polarity [48]. Hydrogen bond strength increases as local polarity decreases, and the non-polar environment is more conducive to the formation of hydrogen bonding. Conversely, water tends to break the originally established hydrogen-bonds network of DES while reforming new hydrogen bonds with DES components. Taking the nano-dispersion made by DES (4:6) in IPM as an example, the morphology observed by TEM was spherical-like particles with nanometer size (Fig. 5E), which was around 5–10 nm and basically consistent with the DLS result. The dependence of conductivity on the mass fraction of DES (4:6) in IPM was shown in Fig. 5F. The curve could be divided into three stages, similar to the phase transition of the microemulsion [17]. The < 45% of the increase region, 45–59% of the increase region, and > 59% of the decrease region corresponded to DES-in-oil, bicontinuous (BC), and oil-in-DES, respectively. When the mass fraction of DES in IPM was < 59%, OMT tended to gather and became a hydrophilic core, while hydrophobic LA with similar properties to IPM became the shell, the core/shell structure DES-RM subsequently self-assembled to form in the IPM without surfactant/co-surfactant.

**Fig. 6:**
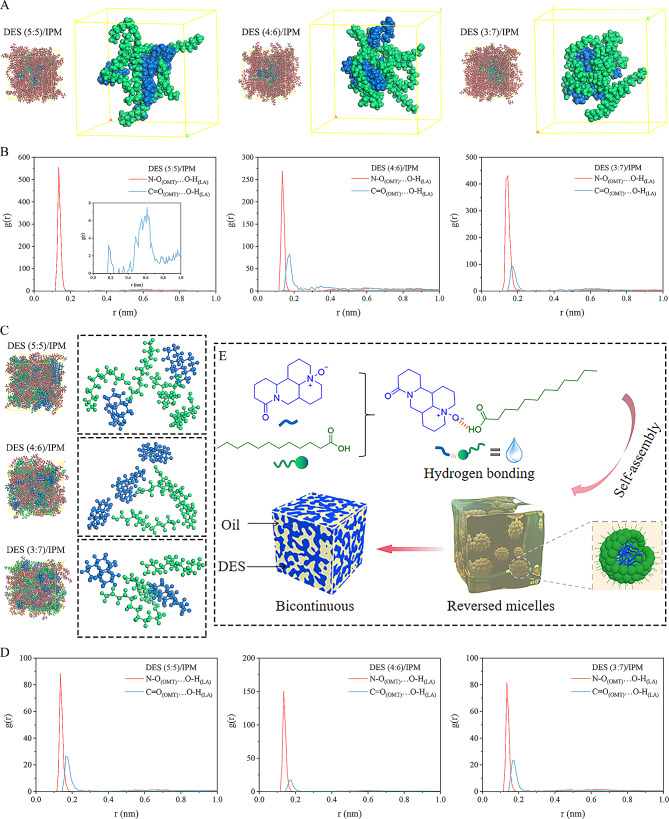
Molecular dynamic assembly of DESs. (**A**) Final trajectory frames and molecular aggregates of 10% DESs; (**B**) Radial distribution function (RDF) curves for specific atoms among 10% DESs; (**C**) Final trajectory frames of 50% DESs and representative snapshots of molecular interaction patterns; (**D**) Radial distribution function (RDF) curves for specific atoms among 50% DESs; (**E**) Schematic diagram of the self-assembly. (blue: OMT; green: LA; brownish yellow: IPM)

The self-assembly process of DESs was also visualized by MD simulation. The aggregation behavior of three DESs in final trajectory frames was presented in Fig. 6. As expected, DESs self-assembled to a nanocluster structure at a low concentration. This nanocluster structure was possibly easier to form as the OMT/LA molar ratio decreased since the molecular area of OMT exposed to the non-polar environment gradually decreased (Fig. 6A), consistent with the lowest CRMC of DES (3:7) of 8 mg/g. To further investigate the driving forces during the self-assembly of DES to RM, a quantitative analysis of hydrogen bond interactions between OMT and LA molecules was carried out. Figure 6B shows the results for RDF, g (r), between specific atoms of OMT and LA. It was obvious that there was a strong correlation between the nitroso of OMT and the carboxyl group of LA at 0.135–0.145 nm, indicating that charge-assisted hydrogen bonding might be the main driving force for self-assembly. Meanwhile, there was also a correlation between the carbonyl group of OMT and the carboxyl group of LA at 0.165–0.175 nm for DES (4:6) and DES (3:7) systems. This was due to the excess LA encircling the OMT carbonyl group. When the proportion of IPM was equal to DESs, a bicontinuous microstructure was formed in the DESs and oil continuous phase with a “worm-like” structure (Fig. 6C). The final snapshot presented the distribution of polar and non-polar domains, in which oil molecules were dispersed in crevices surrounding the hydrophobic regions or, mainly, near LA molecules. Similarly, RDF analysis showed the presence of hydrogen bond interactions between LA and OMT molecules (Fig. 6D).

Through a series of representations and MD simulations, we reasonably speculate the self-assembly mechanism of DESs (Fig. 6E). Firstly, OMT and LA form the complex units that makeup DESs by electrostatic and hydrogen bond interactions. Subsequently, OMT molecules tend to aggregate into clusters in the non-polar environment due to the solvophobic effect. However, the strong molecular interactions among DES components can act as a ‘‘glue” to bond the head groups of OMT and LA together. Hence, these chain aggregates are further stacked through molecular van der Waals forces to form the core/shell structure with an inner hydrophilic core and outer hydrophobic shell. Further, the increase in DES concentration will increase the probability of RM particle collisions and attractions, leading to promote the formation of conductive chains. These chains will be closely connected and fused to each other to form a bicontinuous structure.

### Skin penetration studies

**Fig. 7:**
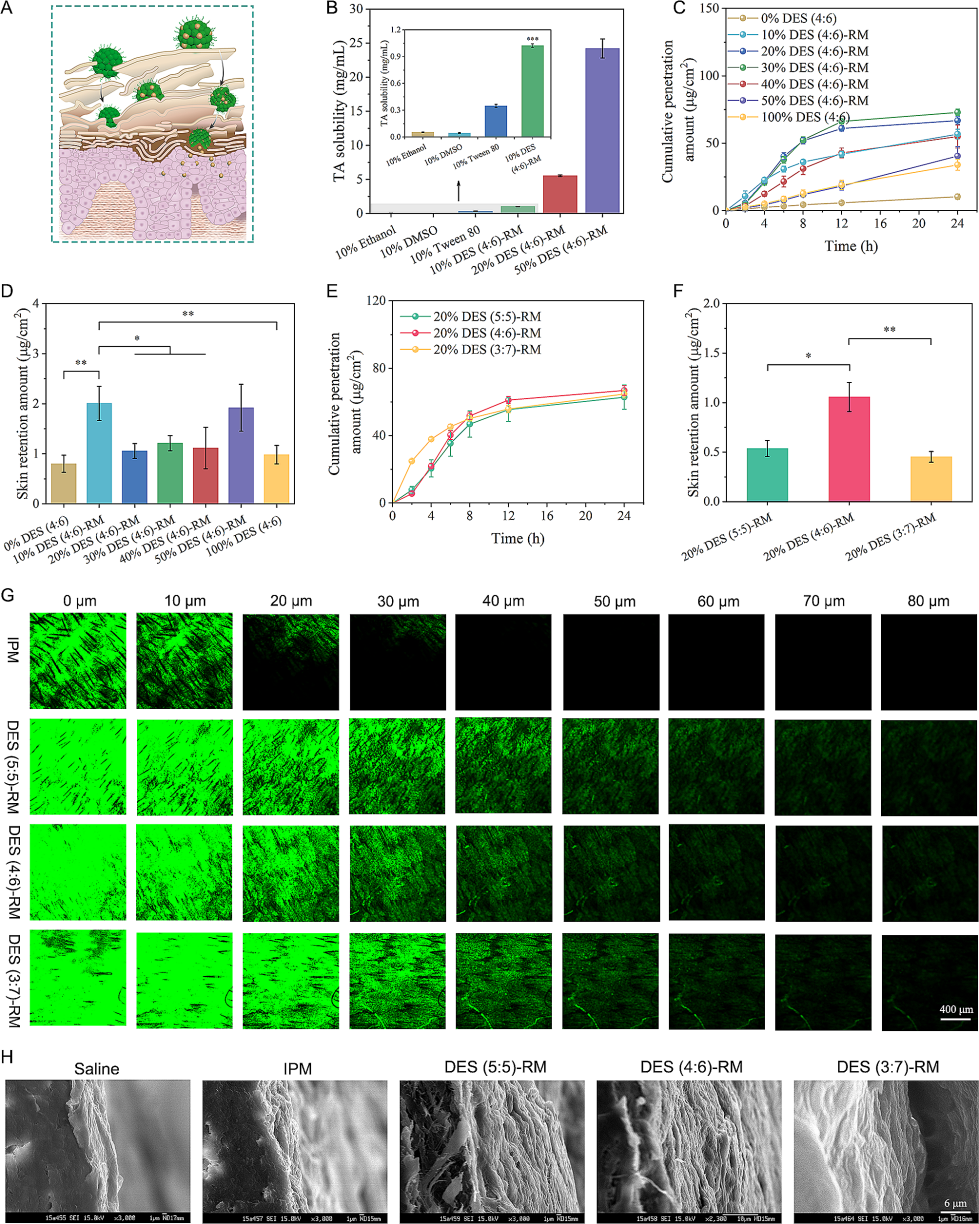
In vitro skin permeation studies. (**A**) Schematic of transdermal delivery of the drug via RM; (**B**) TA solubility in different medium; (**C** and **D**) Effect of DES-RM systems at different concentrations on TA penetration and retention; (**E** and **F**) Effect of DES-RM systems at different mole ratios of OMT/LA on TA penetration and retention; (**G**) Skin penetration depth of coumarin 6 (C6) under incubation with DES-RM systems at different mole ratios of OMT/LA using confocal laser scanning microscopy (CLSM) visualization; (**H**) Scanning electron microscope (SEM) images of stratum corneum longitudinal section after incubation with DES-RM systems at different mole ratios of OMT/LA. ^*^P < 0.05, ^**^P < 0.01, and ^***^P < 0.001

Based on the result of solubility study, we chose TA as a model drug and further investigated the potential of DES-RM for transdermal drug delivery (Fig. 7A). Initially, we evaluated TA solubility in DES (4:6)-RM. TA was dissolved in DES (4:6) and the mixtures were then added to IPM to equilibrate for 24 h. The results showed that the solubility of TA in DES (4:6)-RM was higher than that of traditional pharmaceutically approved solvents such as ethanol, DMSO, and Tween 80 (Fig. 7B). The good solubilizing ability should be due to the molecular interactions between OMT and TA, which facilitated more TA entrapment and adsorption at the cores of RM or the DES/oil interface, as supported by the ^1^H NMR spectra and MD simulation (Additional file 6). Among them, TA-loaded 10% DES (4:6)-RM reached the concentration of commercial TA formulations, and the loading content and loading efficiency were 1.07 ± 0.04% and 93.00 ± 3.65%, respectively. The stability of RM is important for drug delivery. After being frozen and thawed three times, DES (4:6)-RM at different concentrations showed no drug precipitation and phase separation, and no significant change in the size was observed (Additional file 7), indicating that the systems were stable.

Then, the skin penetration curves of TA from different DES (4:6)-RM formulations containing different concentration of DES were assessed (Fig. 7C). The cumulative penetration amounts from low concentration to high concentration of DES (4:6)-RM (0/total IPM, 10%, 20%, 30%, 40%, 50%, 100%/total DES) were 10.37 ± 1.96, 56.83 ± 3.64, 66.83 ± 2.89, 72.96 ± 2.46, 54.99 ± 8.53, 40.49 ± 6.97, and 33.99 ± 4.07 µg/cm^2^, respectively. Compared with IPM and DES alone, DES (4:6)-RM significantly improved the skin penetration of TA. The penetration enhancement was probably attributed to the following reasons. First, the high solubility of TA in RM benefited its permeation across the skin in a passive diffusion manner [49]. The increase in the thermodynamic activity might enlarge the concentration gradient, thereby enhancing drug penetration through the skin. Second, RM had good affinity with the skin lipid layer, therefore, TA encapsulated in RM could easily penetrate the skin through diffusion and fusion. However, the cumulative penetration amount of TA increased as the concentration of DES (4:6) increased below 30%, but then decreased as the concentration of DES (4:6) continued to increase. It is worth noting that DES (4:6)-RM below the concentration of 40% exhibited rapid penetration behavior within 12 h and then slowly penetrated up to 24 h, while 50% DES (4:6)-RM exhibited a linear behavior that approached zero-order within 24 h. This indicated that the RM nanodroplet more helped the drug transport compared with the BC structure. After 24 h of in *vitro* skin penetration, TA retention in the skin was measured (Fig. 7D). The skin retention of 10% DES (4:6)-RM and 50% DES (4:6)-RM was similar and significantly higher than that of IPM and DES alone (*P* < 0.01). In addition, DES (4:6)-RM significantly enhanced TA penetration and retention compared with TA commercial cream (*P* < 0.001, Additional file 8).

The impact of DES-RM at different mole ratios of OMT/LA on TA penetration and skin retention was also assessed. To form RM, the concentration of all DESs was set above CRMC. As shown in Fig. 7E, DES (5:5)-RM, DES (4:6)-RM, and DES (3:7)-RM exhibited similar skin penetration curves at a DES concentration of 20%, with cumulative penetration amounts of 62.90 ± 7.27, 66.83 ± 2.89, and 64.74 ± 2.31 µg/cm^2^. However, from the result of TA retention, DES (4:6)-RM exhibited higher drug retention and was superior to DES (5:5)-RM and DES (3:7)-RM (Fig. 7F). In summary, both transcutaneous penetration and skin retention of TA from DES (4:6)-RM were greatly improved.

Furthermore, DES-RM was loaded with hydrophobic C6 as a fluorescent probe to visualize the penetration depth (Fig. 7G). The fluorescent images revealed that IPM alone only offered relatively low C6 fluorescence intensity and permeation depth (up to 30 μm) in the skin. A large amount of C6 may be embedded in the lipid matrix (0 ~ 10 μm) of SC. In contrast, the skins treated with DES-RM presented a significantly deeper permeation depth (up to 80 μm) with stronger green fluorescence intensity. Such penetration depth was sufficient to reach the active epidermis layer and dermis layer. Consequently, DES-RM delivered the drug more efficiently into the skin in contrast to the oil phase of IPM.

### Skin penetration mechanism

The skin penetration enhancement has three possible mechanisms: (1) lipid extraction and lipid fluidization [6]; (2) interaction with intracellular keratin [50]; (3) increasing partitioning into the skin [49]. These mechanisms are mainly related to the “brick and mortar” structure of SC, which is usually considered the main rate-limiting step to drug absorption through the skin. Hence, we evaluated the effect of DES-RM on the structure of SC by SEM and FTIR. Figure 7H shows the microstructure images of the SC after treatment with DES-RM formulations. Normal skin treated with normal saline was used as a control. After incubation with IPM alone or DES-RM, it was found distinct gaps between the multilayer lipids of the SC. This indicated that the lamellar pattern arrangement of SC lipids within the intercellular region may be disrupted, which would lead to an increase in fluidity [11]. Moreover, the exfoliation of DES-RM on the lamellar pattern of SC was even more significant in comparison with IPM alone, suggesting that RM could increase drug permeation by creating greater fluidity or more kinks in the SC lipids.

Next, we tested how DES-RM formulations interacted with the lipid and keratin of SC by FTIR spectroscopy. By self-contrast, we compared FTIR spectra of the SC before and after treatment (Table 2). In the case of untreated skin samples, the characteristic peaks of the lipid were located at 2918 (CH_2_ asymmetric vibration) and 2850 cm^− 1^ (CH_2_ symmetric vibration), and the characteristic peaks of keratin were located at 1645 (amide I) and 1545 cm^− 1^ (amide II). However, all the treated skin samples showed obvious peak shifts for both the lipid and keratin. For the lipid matrix, the CH_2_ asymmetric and symmetric stretching vibrations shifted to a higher wavenumber, which corresponded to a transformation of the lipid from an orthorhombic conformation to a liquid-crystalline structure, presenting a more fluid structure [51]. Moreover, FTIR peak areas which are indicators of the SC lipid content were deconvoluted by considering peaks Gaussian with several iterations to facilitate a spectrum comparison before and after incubation. As shown in Additional file 9, the peak areas of both CH_2_ asymmetric vibration and symmetric vibration slightly increased after incubation. This could be attributed to the lipophilicity of IPM or LA, causing them to remain embedded in the lipid matrix of the SC. IPM, a conventional penetration enhancer, can act on the SC, permeate into the lipid bilayer, and increase the fluidity of membranes [52]. However, DES-RM resented more significant fluidization on the SC compared with IPM alone because of the synergistic enhancing effect of multiple components in the system. Additionally, amide I and amide II bands of keratin showed evident blue shifts for DES-RM, which indicated that the conformation of keratin converted from an organized α-helical to a randomly coiled structure by treatment with DES-RM [50]. In summary, DES-RM enhanced transcutaneous absorption of the drug through lipid fluidization and de-keratinization of corneocytes.

**Table 2:** Changes in FTIR spectra of skin samples before and after treatment of DES-RM systems at different mole ratios.^*a*^

Name	Treatment	Lipid				Keratin			
		CH_2_.Asymmetric (cm ^− 1^)	ΔShift	CH_2_.Symmetric (cm ^− 1^)	ΔShift	Amide I (cm ^− 1^)	ΔShift	Amide II (cm ^− 1^)	ΔShift
IPM	Before	2918.32 ± 0.08	+ 3.40	2850.32 ± 0.01	+ 1.99	1644.91 ± 0.71	−2.27	1545.24 ± 0.26	−9.44
	After	2921.72 ± 0.46*		2852.31 ± 0.23*		1642.64 ± 0.58		1535.80 ± 0.28*	
20% DES (5:5)-RM	Before	2919.14 ± 1.00	+ 4.44	2850.66 ± 0.47	+ 3.07^#^	1645.64 ± 0.20	+ 3.08	1545.20 ± 0.10	+ 5.54
	After	2923.58 ± 0.39*		2853.73 ± 0.34*		1648.72 ± 0.54*		1550.74 ± 0.67*	
20% DES (4:6)-RM	Before	2918.18 ± 0.05	+ 5.70^#^	2850.26 ± 0.04	+ 3.80^#^	1644.27 ± 0.14	+ 4.06	1545.14 ± 0.12	+ 5.32
	After	2923.88 ± 0.06*		2854.06 ± 0.06*		1648.33 ± 0.37*		1550.46 ± 0.02*	
20% DES (3:7)-RM	Before	2918.46 ± 0.58	+ 5.20	2850.38 ± 0.31	+ 3.60^#^	1644.96 ± 0.64	+ 2.51	1545.24 ± 0.44	+ 3.56
	After	2923.66 ± 0.08*		2853.98 ± 0.03*		1647.47 ± 1.50		1548.80 ± 0.15*	

^*a*^ DES-RM systems application remarkably increased the fluidity of SC. ^*^P < 0.05, compared with untreated skin samples. ^#^*P* < 0.05, compared with IPM

### In vivo therapeutic efficacy

The therapeutic potential of TA@DES-RM was further evaluated using an IMQ-induced mouse model of psoriasis. First, the biocompatibility of TA@DES-RM was assessed (Additional file 10). The skin appearance, body weight, and blood biochemical indicators of the mice in low-dose and high-dose groups showed no obvious difference compared with the control group throughout the experiment. Moreover, H&E staining of major organs of the mice did not show obvious tissue injuries. These results demonstrated the outstanding biocompatibility of TA@DES-RM.

**Fig. 8:**
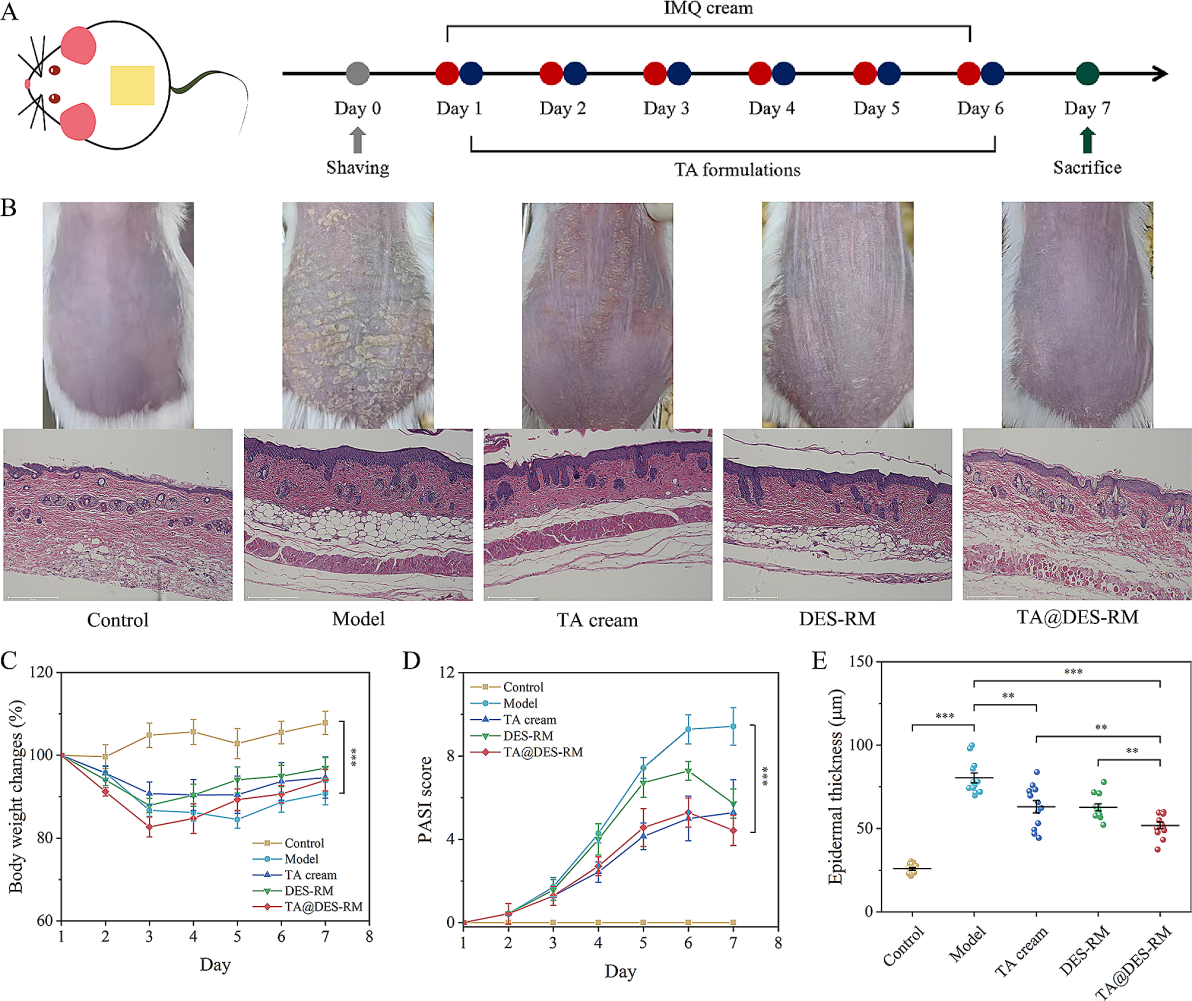
Transdermal administration of TA@DES-RM alleviated psoriasis-like symptoms. (**A**) Experimental timeline; (**B**) Representative photographs and H&E staining of the mice skin on day 7; (**C**) The body weight change of mice during the treatment; (**D**) PASI score reflecting the severity of psoriasis; (**E**) Epidermal thickness measured by H&E staining sections. Analysis was conducted in three micrographs of different areas in each skin sample. ^*^P < 0.05, ^**^P < 0.01, and ^***^P < 0.001

To establish the psoriatic mouse model, IMQ cream was topically administered on the back skin for 6 consecutive days inducing typical psoriatic manifestations, such as erythema, scales, and thickening (Fig. 8A and B). In the presence of treatment, all formulations relieved IMQ-induced psoriasis-like dermatitis. Representative photos of dorsal skin on day 7 displayed the most effective inhibitory effect of TA@DES-RM on psoriatic symptoms (Fig. 8B). Compared with the model group, mice in the TA@DES-RM group recovered most of the lost weight on day 7 after treatment (Fig. 8C). Moreover, the PASI score of TA@DES-RM group was significantly lower than that of the model group (*P* < 0.001, Fig. 8D), indicating the therapeutic effect of TA@DES-RM on psoriasis. No significant differences were observed for body weight change and PASI score between TA cream and TA@DES-RM groups. Nevertheless, the results of H&E staining showed that the epidermis thickness of mice in the TA@DES-RM group was significantly less than that of the TA cream group (*P* < 0.01, Fig. 8E). Specifically, the epidermis thickness of the mice in the TA cream, DES-RM, and TA@DES-RM groups on day 7 was reduced from 80.38 ± 9.76 to 63.07 ± 12.06, 62.73 ± 7.01, and 51.88 ± 6.75 μm, respectively. This suggested that the application of TA@DES-RM had a more effective inhibitory effect on keratinocyte proliferation than using TA cream. Notably, blank DES-RM also relieved the topical psoriasis manifestations, which was attributed to the anti-inflammatory effect of DES itself. It is reported that OMT can inhibit IMQ-induced pruritus and alleviate keratinization of skin and inflammatory infiltration [53]; meanwhile, LA also exerts certain anti-inflammatory activity [54]. Therefore, DES as an adjuvant of TA is beneficial for psoriasis treatment.

**Fig. 9:**
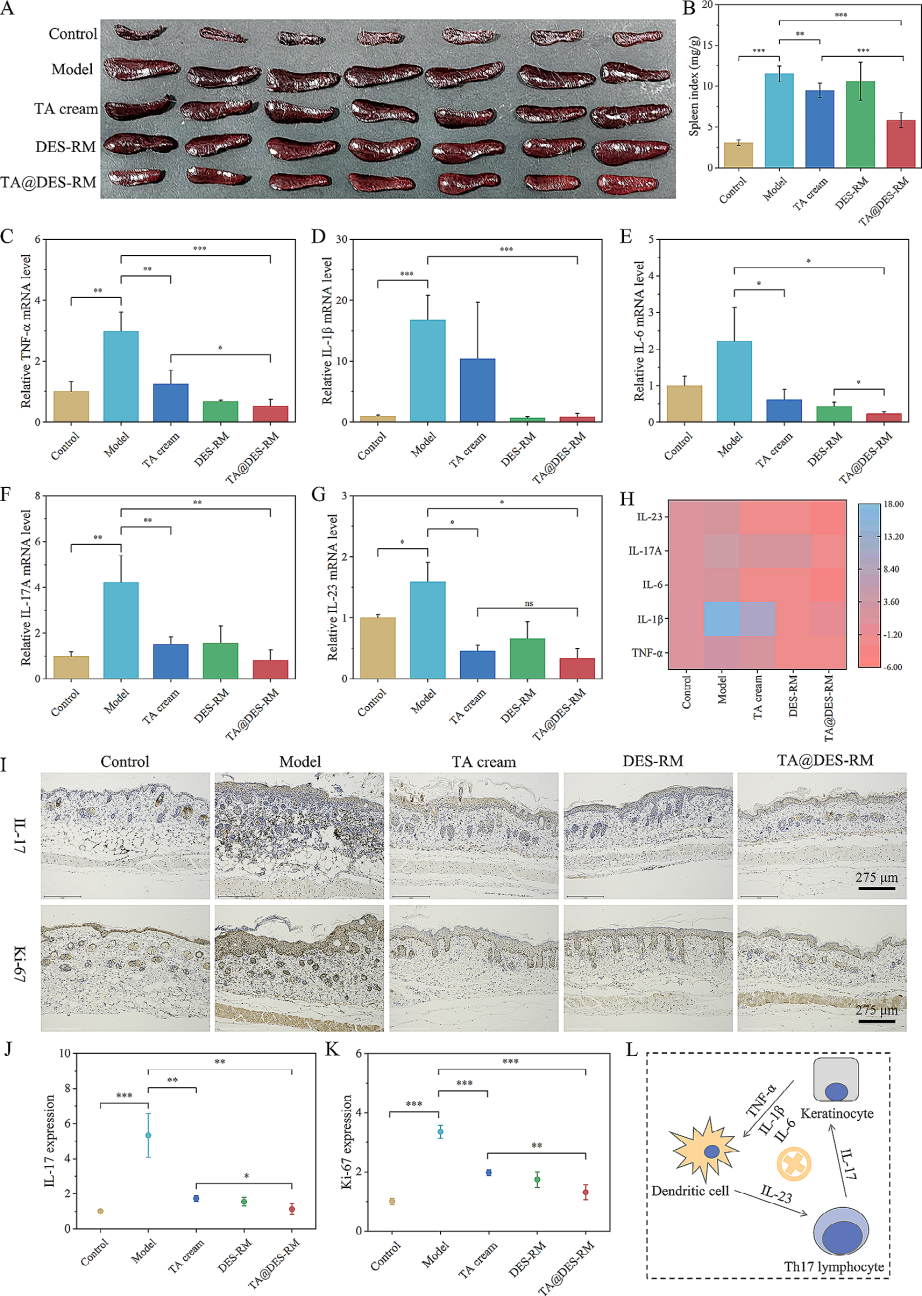
TA@DES-RM relieved IMQ-induced skin inflammatory responses. (**A**) Representative images of mouse spleen on day 7; (**B**) spleen index calculated by spleen weight/body weight (mg/g); (**C**-**G**) Relative mRNA expression levels of pro-inflammatory factors in mouse skin (TNF-α, IL-1β, IL-6, IL-17 A, and IL-23); (**H**) Heat map of pro-inflammatory factors quantified by qPCR normalized to the control group; (**I**) Immunohistochemical staining of IL-17 and Ki-67 in the skin. (**J** and **K**) IL-17 and Ki-67 expression assessed by Image J software; (**L**) Schematic diagram of the mechanism of using TA@DES-RM to treat psoriasis. ^*^P < 0.05, ^**^P < 0.01, and ^***^P < 0.001

Psoriasis is a chronic inflammatory-mediated skin disease. Dysregulated innate and adaptive immune responses are major contributors to the development of psoriasis [55]. Thus, we further examined its efficacy in the treatment of psoriasis-like skin dermatitis. The spleen is a critical immune organ, and the spleen index reflects the level of immune function [56]. After the experiment, the spleen was collected from each mouse. The spleen of the model group was remarkably enlarged compared with the control group. Although the spleen size and spleen index in the TA@DES-RM group were larger than those in the control group, they were significantly smaller than those in the model group (*P* < 0.001, Fig. 9A and B). The increase in the spleen index is commonly associated with inflammation and immune activation. TA encapsulated in RM exhibited better transdermal absorption and combined with the anti-inflammatory activity of DES at the same time, thereby significantly suppressing IMQ-induced inflammatory storms. Furthermore, we also measured the relative mRNA expression levels of related pro-inflammatory factors known as markers to psoriasis by qRT-PCR. Consistent with the above results, the expression levels of inflammatory factors of the model group increased significantly, which was inhibited by each group of treatment and most significantly by the TA@DES-RM group, followed by DES-RM and TA cream groups (Fig. 9C-F). From the heat map of pro-inflammatory factors normalized to the control group, TA@DES-RM downregulated the expression levels of TNF-α, IL-1β, IL-6, IL-17 A, and IL-23, but TA cream and DES-RM only downregulated the expression levels of TNF-α, IL-6, and IL-23 (Fig. 9H). These results suggest that TA@DES-RM can better alleviate pro-inflammatory effects and relieve psoriasis symptoms. It is known that the IL-23/Th 17 pathway is the core axis leading to the pathogenesis of psoriasis [55], we next examined the expression patterns of IL-17 in the skin. Immunohistochemistry showed that TA@DES-RM significantly suppressed the expression of IL-17 in psoriasis-like skin, comparable to normal skin levels (Fig. 9I and J). Additionally, keratinocyte proliferation significantly decreased in the TA@DES-RM group reflected by a reduction in Ki-67 (a nuclear protein closely related to cell proliferation) expression (Fig. 9I and K). These were consistent with the above results of H&E staining and qRT-PCR. The anti-inflammatory effect of TA@DES-RM on psoriatic mice was summarized in Fig. 9L.

Together, three major advantages for anti-psoriasis in transdermal delivery can be gained by this simple self-assembly system. First, high drug solubility in DES provides a good foundation for drug diffusion. Second, RM, the nanocarrier, both promotes drug penetration into deep skin and increases retention in the skin. Third, DES-RM not only can serve as a carrier for drug delivery but may also exert a certain adjunct therapeutic efficacy.

## Conclusions

In conclusion, a self-assembly RM without surfactant/co-surfactant based on DESs formulated by OMT and LA was fabricated for transdermal delivery of sparingly soluble drugs. Through the constructed QSAR model, the solubility of various drugs in DESs could be preliminarily estimated. All experiments combined with MD simulations demonstrated that OMT and LA molecules were connected by hydrogen bond and electrostatic interactions. In the oil phase, the chain aggregates were further stacked under weak van der Waals forces to form the RM structure with an inner hydrophilic core (OMT) and an outer hydrophobic shell (LA). Taking TA as a model drug, the skin penetration studies suggested that DES-RM delivered a significantly higher amount of TA transdermally and topically by increasing the lipid fluidity of SC and affecting keratin conformation. Finally, the biocompatibility and therapeutic efficacy of TA@DES-RM were further verified in the IMQ-induced psoriasis-like mouse model. Therefore, this DES-RM provides a promising strategy for the development of an efficient transdermal delivery system for sparingly soluble drugs, and computation simulation is a companionship approach to comprehend the molecular mechanisms behind DES and DES-RM performance and aid future formulation design.

### Electronic supplementary material

Below is the link to the electronic supplementary material.

Supplementary Material 1

Supplementary Material 2

Supplementary Material 3

Supplementary Material 4

Supplementary Material 5

Supplementary Material 6

Supplementary Material 7

Supplementary Material 8

Supplementary Material 9

Supplementary Material 10

## Data Availability

Data is provided within the manuscript or supplementary information files, and data will be made available on request.
