# Supplementary material for: Deep eutectic solvent self-assembled reverse nanomicelles for transdermal delivery of sparingly soluble drugs
Source: J Nanobiotechnology. 2024 May 21;22:272. doi: 10.1186/s12951-024-02552-y (PMC11106993; doi:10.1186/s12951-024-02552-y)
Supplement: Supplementary file 1 — Supplementary Material 1 [file 12951_2024_2552_MOESM1_ESM.doc]

1. **Quantum chemical calculation**

Quantum chemical calculation was performed with the Gaussian 16 program package. The geometry optimizations of dimeric OMT-OMT, LA-LA, and OMT-LA were carried out with the B3LYP/6-31G(d,p) basis set. All the optimized structures were confirmed to be minimum energy conformations by harmonic vibrational frequency calculation (the absence of any negative frequencies). The interaction energy (Δ*E*) was calculated by considering the difference between the total energy (*E*_dimer_) of the dimer and the sum of the energies (*E*_monomer_) of the corresponding monomers and corrected using the GD3BJ correction method to include the dispersion effects on the structures. Meanwhile, basis set superposition error (BSSE) correction was implemented via the procedure. The corrected interaction energy is calculated as:

$$\Delta E=E_{\dim er}-\sum E_{monomer}+E_{BSSE}$$

To better understand the interaction types and strength between OMT and LA inside DES, we carried out the molecular electrostatic potential (ESP) analysis and the independent gradient model based on Hirshfeld partition (IGMH) by using the Multiwfn 3.8 program [1-3].





Figure S2. Quantitative distribution of ESP of OMT (A), LA (B), and OMT-LA (C).

**References**

1. T. Lu, F. Chen, Multiwfn: A multifunctional wavefunction analyzer, Journal of Computational Chemistry, 33 (2012) 580-592.

2. S. Manzetti, T. Lu, The geometry and electronic structure of Aristolochic acid: possible implications for a frozen resonance, Journal of Physical Organic Chemistry, 26 (2013) 473-483.

3. T. Lu, Q. Chen, Independent gradient model based on Hirshfeld partition: A new method for visual study of interactions in chemical systems, Journal of Computational Chemistry, 43 (2022) 539-555.
