# Supplementary material for: Deep eutectic solvent self-assembled reverse nanomicelles for transdermal delivery of sparingly soluble drugs
Source: J Nanobiotechnology. 2024 May 21;22:272. doi: 10.1186/s12951-024-02552-y (PMC11106993; doi:10.1186/s12951-024-02552-y)
Supplement: Supplementary file 2 — Supplementary Material 2 [file 12951_2024_2552_MOESM2_ESM.doc]

1. **MD simulation of DESs**

MD simulation was carried out according to previously reported by Gupta et al, with slight modifications [1,2]. The molecular structures of OMT and LA were obtained from the PubChem Database. For DES (6:4), DES (5:5), DES (4:6), and DES (3:7), amorphous models consisting of 60/40, 50/50, 40/60, and 30/70 molecules were generated at 298 K. The dimensions of initial systems with periodic boundary conditions were 3.41 × 3.41 × 3.41, 3.38 × 3.38 × 3.38, 3.35 × 3.35 × 3.35, and 3.32 × 3.32 × 3.32 nm, respectively. Energy minimization of each system was carried out using the steepest descent and conjugate gradient methods. Following minimization, the MD simulation was carried out in two phases: the equilibration phase (500 ps of isothermal (NVT) simulation) and the production phase (500 ps of isobaric-isothermal (NPT) simulation). The final structure from each MD run was used as the starting structure for the subsequent run. The Berendsen thermostat and barostat was used to maintain the temperature (298 K) and pressure (1 atm), with a time step of 1 fs. The van der Walls interaction was calculated using atom-based summation with a cut-off distance of 1.25 nm, while the electrostatic interaction used Ewald summation. A total of 100 trajectory frames were captured during the production run for computing the radial distribution function (RDF) and solubility parameter (*δ*). With respect to the geometry of the hydrogen bond definition, the distance between the hydrogen atom and the hydrogen bond receptor was less than 0.35 nm, and the angle was ≥ 120°.


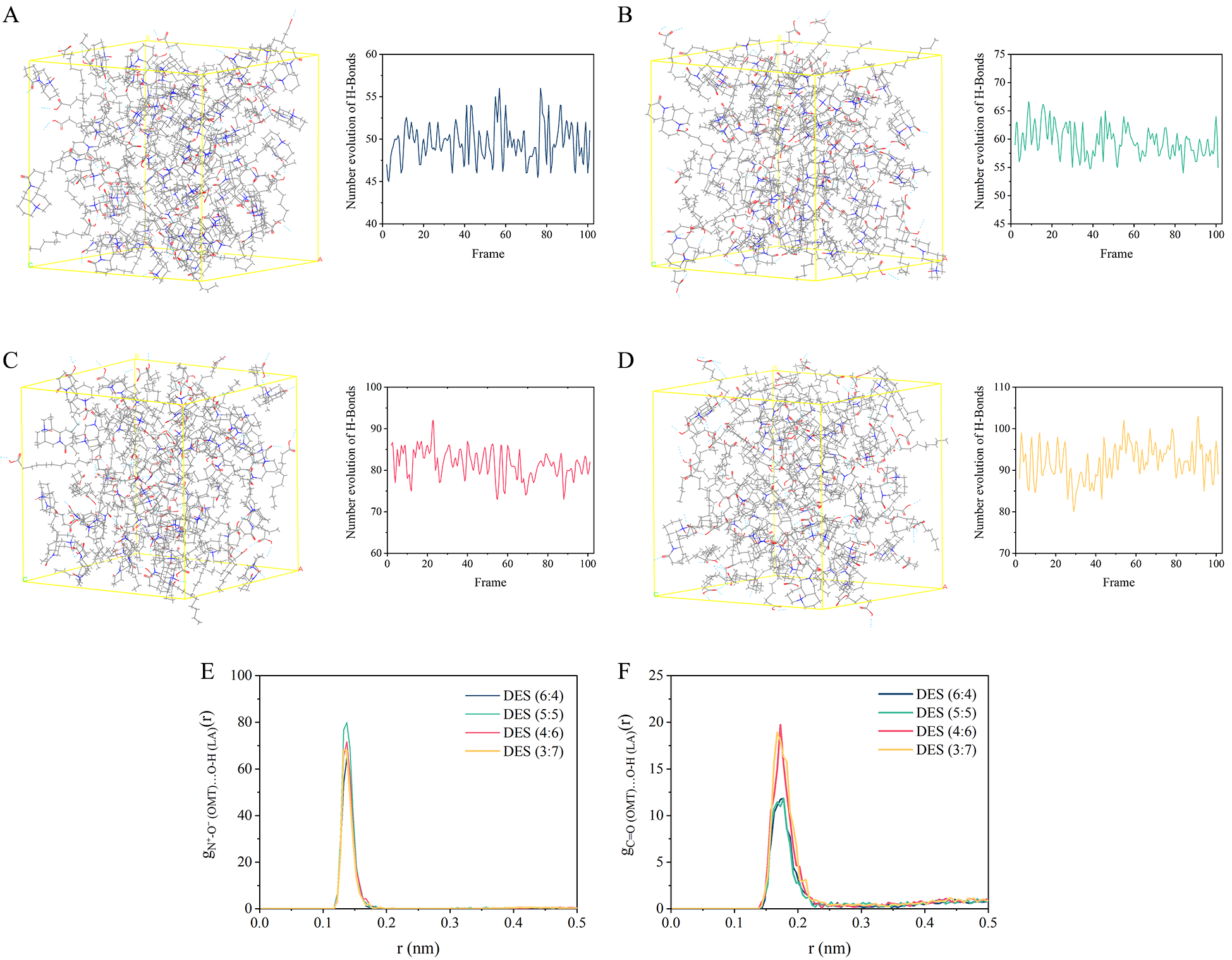


Figure S1. Final trajectory frame and hydrogen bond statistics for (A) DES (6:4), (B) DES (5:5), (C) DES (4:6), and (D) DES (3:7) resulting from the production run. (E) RDF analysis between the O atom at the nitroso group of OMT and the H atom at the carboxyl group of LA. (F) RDF analysis between the O atom at the carbonyl group of OMT and the H atom at the carboxyl group of LA.

**References**

1. J. Gupta, C. Nunes, S. Jonnalagadda, A Molecular Dynamics Approach for Predicting the Glass Transition Temperature and Plasticization Effect in Amorphous Pharmaceuticals, Molecular Pharmaceutics, 10 (2013) 4136-4145.

2. J. Gupta, C. Nunes, S. Vyas, S. Jonnalagadda, Prediction of Solubility Parameters and Miscibility of Pharmaceutical Compounds by Molecular Dynamics Simulations, The Journal of Physical Chemistry B, 115 (2011) 2014-2023.
