# Supplementary material for: Deep eutectic solvent self-assembled reverse nanomicelles for transdermal delivery of sparingly soluble drugs
Source: J Nanobiotechnology. 2024 May 21;22:272. doi: 10.1186/s12951-024-02552-y (PMC11106993; doi:10.1186/s12951-024-02552-y)
Supplement: Supplementary file 3 — Supplementary Material 3 [file 12951_2024_2552_MOESM3_ESM.doc]

1. **The high performance liquid chromatography (HPLC) conditions**

Table S1. The liquid chromatography conditions of various drugs. The same conditions (column temperature: 30 °C, flow rate: 1 mL/min) were used, unless stated otherwise.

| Name | Mobile phase |
| --- | --- |
| QUE | methanol:0.2% phosphoric acid aqueous solution = 60:40 (v/v), UV: 360 nm |
| BAI | methanol:0.2% phosphoric acid aqueous solution = 47:53 (v/v), UV: 280 nm |
| ADA | acetonitrile: tetrahydrofuran: 2% trifluoroacetic acid aqueous solution = 42:32:26 (v/v), UV: 235 nm |
| TA | methanol:water = 65:35 (v/v), UV: 240 nm |
| PIR | acetonitrile:phosphate buffer (0.01 M potassium dihydrogen phosphate aqueous solution and adjusted to pH 2.5) = 35:65 (v/v), UV: 230 nm |
| FA | methanol:1% glacial acetic acid aqueous solution = 40:60 (v/v), UV: 322 nm |

1. **QSAR analysis**

Part of the molecular descriptors was from PubChem and CAS SciFinder databases, including hydrogen bond donor count (*HBD*), hydrogen bond acceptor count (*HBA*), molar volume (*V*_m_), melting point (*T*_m_), topological polar surface area (*TPSA*), octanol-water partition coefficient (Xlog *P*), and acidity coefficient (*pK*a). The molecular refractivity (*MR*) and solubility parameters of 7 drugs were calculated using Materials Studio 8.0 software. The SDF structures of adapalene (ADA), baicalin (BAI), triamcinolone acetonide (TA), piroxicam (PIR), quercetin (QUE), ferulic acid (FA), and minoxidil (MIN) were obtained from the PubChem Database. The COMPASS force field was utilized for subsequent all calculations. Amorphous models for each drug (50 molecules) were generated at 298 K, with periodic boundary conditions. Energy minimization of each system was carried out using the steepest descent and conjugate gradient methods. MD simulation was carried out in two phases: the equilibration phase (200 ps of isobaric-isothermal (NPT) simulation) and the production phase (200 ps of isothermal (NVT) simulation). Next, we followed the same procedure as Section 1. The data from the final 100 ps was used for computing the average *δ*.

Two QSAR models (genetic function approximation (GFA) and partial least square (PLS)) were generated and analyzed using Materials Studio 8.0 software. All data were normalized by mean/SD to eliminate the influence of variance between different individuals. Descriptors with high cross-correlation values (> 0.7) were not considered. The best models were identified with correlation coefficient (*R*^2^) and cross-validation coefficient (*Q*^2^) and models with up to three descriptors were selected [1].

Table S2. The calculated solubility parameters ((J/cm^3^)^0.5^) using MD.

| Name | Solubility parameter | Name | Solubility parameter |
| --- | --- | --- | --- |
| ADA | 20.316 | DES (5:5) | 22.473 |
| BAI | 26.591 | DES (4:6) | 22.445 |
| TA | 19.903 | DES (3:7) | 21.881 |
| PIR | 26.173 |  |  |
| QUE | 28.683 |  |  |
| FA | 25.980 |  |  |
| MIN | 27.660 |  |  |

Abbreviation: ADA, adapalene; BAI, baicalin; TA, triamcinolone acetonide; PIR, piroxicam; QUE, quercetin; FA, ferulic acid; MIN, minoxidil.

Table S3. The physicochemical descriptors of various drugs.

| Name | log *S* | *HBD* | *HBA* | *MR* | *V*_m_ | *T*_m_ | *TPSA* | Xlog *P* | Δ*SP* | *pK*a |
| --- | --- | --- | --- | --- | --- | --- | --- | --- | --- | --- |
| DES (5:5) | | | | | | | | | | |
| ADA | 1.740 | 1 | 3 | 121.574 | 334.55 | 321 | 46.5 | 7.7 | 2.157 | 4.23 |
| BAI | 1.486 | 6 | 11 | 104.427 | 256.99 | 223 | 183.0 | 1.1 | 4.118 | 2.72 |
| TA | 1.973 | 2 | 7 | 111.739 | 326.69 | 293 | 93.1 | 2.5 | 2.570 | 12.87 |
| PIR | 1.685 | 2 | 5 | 84.976 | 211.96 | 199 | 108.0 | 3.1 | 3.700 | 4.50 |
| QUE | 0.666 | 5 | 7 | 75.428 | 168.00 | 317 | 127.0 | 1.5 | 6.210 | 6.31 |
| FA | 1.899 | 2 | 4 | 51.292 | 147.55 | 169 | 66.8 | 1.5 | 3.507 | 4.58 |
| MIN | 1.292 | 4 | 3 | 52.277 | 137.66 | 248 | 88.9 | 1.2 | 5.187 | 9.39 |
| DES (4:6) | | | | | | | | | | |
| ADA | 1.479 | 1 | 3 | 121.574 | 334.55 | 321 | 46.5 | 7.7 | 2.129 | 4.23 |
| BAI | 1.558 | 6 | 11 | 104.427 | 256.99 | 223 | 183.0 | 1.1 | 4.146 | 2.72 |
| TA | 1.814 | 2 | 7 | 111.739 | 326.69 | 293 | 93.1 | 2.5 | 2.542 | 12.87 |
| PIR | 1.644 | 2 | 5 | 84.976 | 211.96 | 199 | 108.0 | 3.1 | 3.728 | 4.50 |
| QUE | 0.818 | 5 | 7 | 75.428 | 168.00 | 317 | 127.0 | 1.5 | 6.238 | 6.31 |
| FA | 1.881 | 2 | 4 | 51.292 | 147.55 | 169 | 66.8 | 1.5 | 3.535 | 4.58 |
| MIN | 1.509 | 4 | 3 | 52.277 | 137.66 | 248 | 88.9 | 1.2 | 5.215 | 9.39 |

Abbreviation: log *S,* logarithm of solubility; *HBD*, hydrogen bond donor count; *HBA*, hydrogen bond acceptor count; *MR*, molecular refractivity; *V*_m_, molar volume; *T*_m_, melting point; *TPSA*, topological polar surface area; Xlog *P*, octanol-water partition coefficient; *pK*a, acidity coefficient; Δ*SP*, the absolute value of the difference of solubility parameter between DES and drug.





Figure S3. QSAR correlation matrix for (A) DES (5:5) and (B) DES (4:6).





Figure S4. Experimental *vs* predicted plots of the solubility (log *S*) for (A) DES (5:5) and (B) DES (4:6).

The best equations based on PLS analysis were as follow:

DES (5:5): $\log S=-0.1578T_{m}-0.4671\Delta SP+0.1076pKa+1.5223$

*R*^2^=0.9927, *Q*^2^=0.4140, *RMSE*=0.4693

DES (4:6): $\log S=-0.1978T_{m}-0.3213\Delta SP+0.0653pKa+1.4707$

*R*^2^=0.9666, *Q*^2^=0.4812, *RMSE*=1.0016

**References**

1. B. Giner, C. Lafuente, D. Lapeña, D. Errazquin, L. Lomba, QSAR study for predicting the ecotoxicity of NADES towards Aliivibrio fischeri. Exploring the use of mixing rules, Ecotoxicology and Environmental Safety, 191 (2020).
