# Supplementary material for: Deep eutectic solvent self-assembled reverse nanomicelles for transdermal delivery of sparingly soluble drugs
Source: J Nanobiotechnology. 2024 May 21;22:272. doi: 10.1186/s12951-024-02552-y (PMC11106993; doi:10.1186/s12951-024-02552-y)
Supplement: Supplementary file 4 — Supplementary Material 4 [file 12951_2024_2552_MOESM4_ESM.doc]

1. **Characterization of monomer components**





Figure S5. Characterization of OMT and LA. DSC thermograms of (A) OMT and (B) LA. TGA/DTG curves of (C) OMT and (D) LA. FTIR spectra of (E) OMT and (F) LA. ^1^H NMR spectra (400 MHz, DMSO-*d_6_*) of (G) OMT and (H) LA.

Table S4. Decomposition temperatures of DESs derived from DTG curves.

| Name | | *T*_onset_ /°C | *T*_peak_ /°C |
| --- | --- | --- | --- |
| monomers | OMT | 270 | 284 |
|  | LA | 146 | 205 |
| DESs | DES (6:4) | 178 | 217 |
|  | DES (5:5) | 175 | 223 |
|  | DES (4:6) | 173 | 222 |
|  | DES (3:7) | 167 | 212 |

1. **Rheological measurement of supramolecular hydrogel formed by DES**

Rheological measurements were performed on the MCR 302e rotational rheometer (Anton Paar, Austria) using a cone-plate geometry (4°/20 mm). Initially, a resting time of 120 s at room temperature was used to eliminate stresses produced by the sample loading. The strain sweep was performed at an angular frequency of 10 rad/s in a strain range from 0.001 to 10%. The viscosity was measured with the shear rate varying from 0.01 to 100 s^-1^.

The elastic module (G′) was higher than the viscous module (G″) in the low-strain region (< 0.69%), which suggested the formation of a gel. The strong shear-thinning phenomenon indicated the typical non-Newtonian fluid behavior.


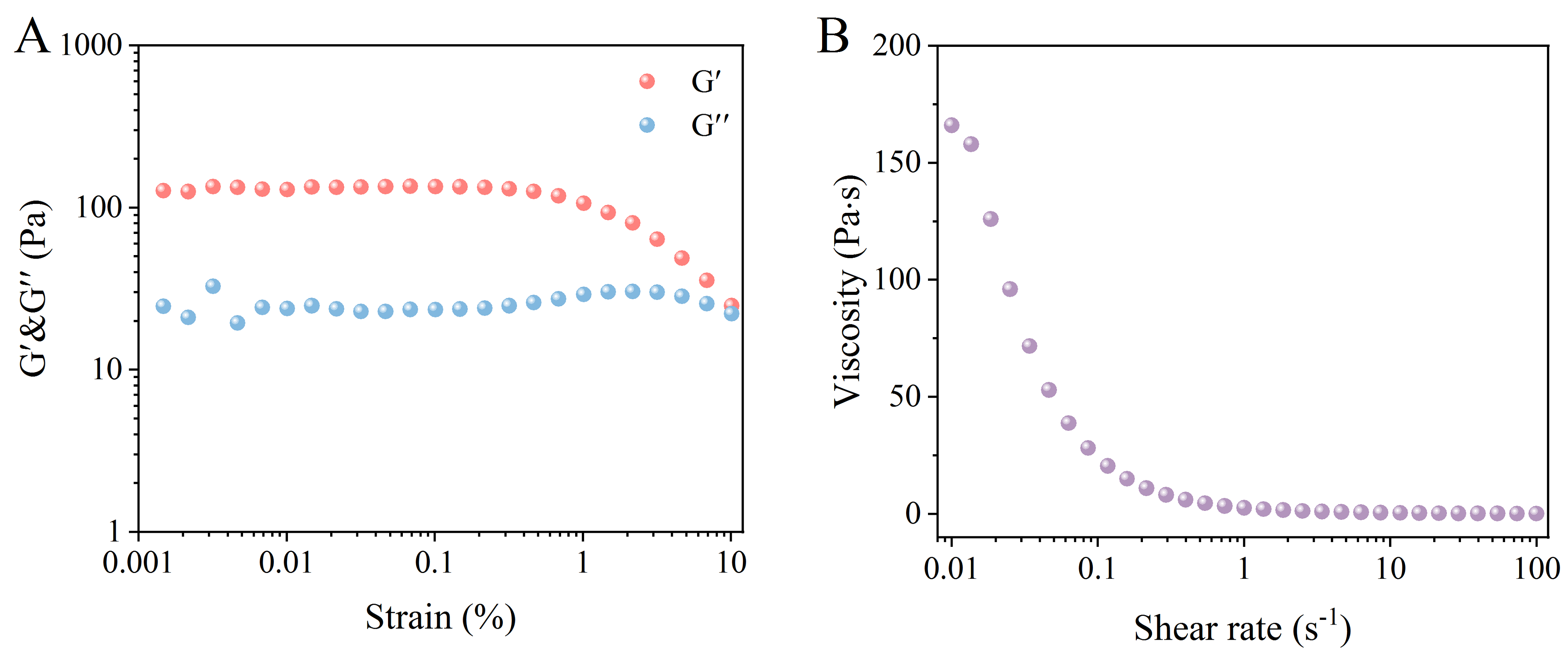


Figure S6. (A) The strain sweep measurement of the DES (4:6) + water mixture (40:60, w/w). (B) The plot of the viscosity of the DES (4:6) + water mixture (40:60, w/w) against shear rate.
