# Supplementary material for: Deep eutectic solvent self-assembled reverse nanomicelles for transdermal delivery of sparingly soluble drugs
Source: J Nanobiotechnology. 2024 May 21;22:272. doi: 10.1186/s12951-024-02552-y (PMC11106993; doi:10.1186/s12951-024-02552-y)
Supplement: Supplementary file 5 — Supplementary Material 5 [file 12951_2024_2552_MOESM5_ESM.doc]

1. **FTIR spectra of DESs in IPM or water**


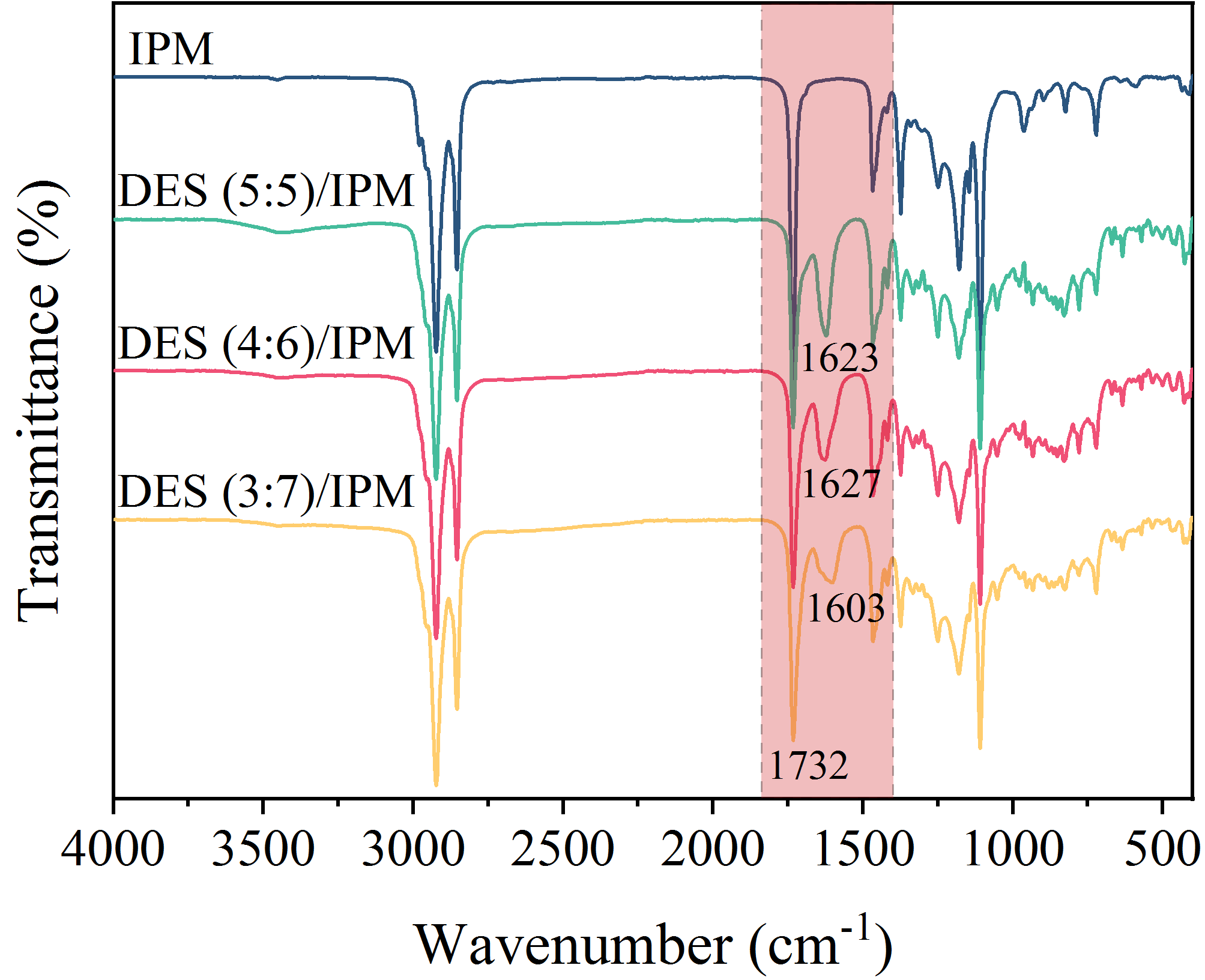


Figure S7. FTIR spectra of DESs in IPM. The concentration of DESs was 50 wt%.


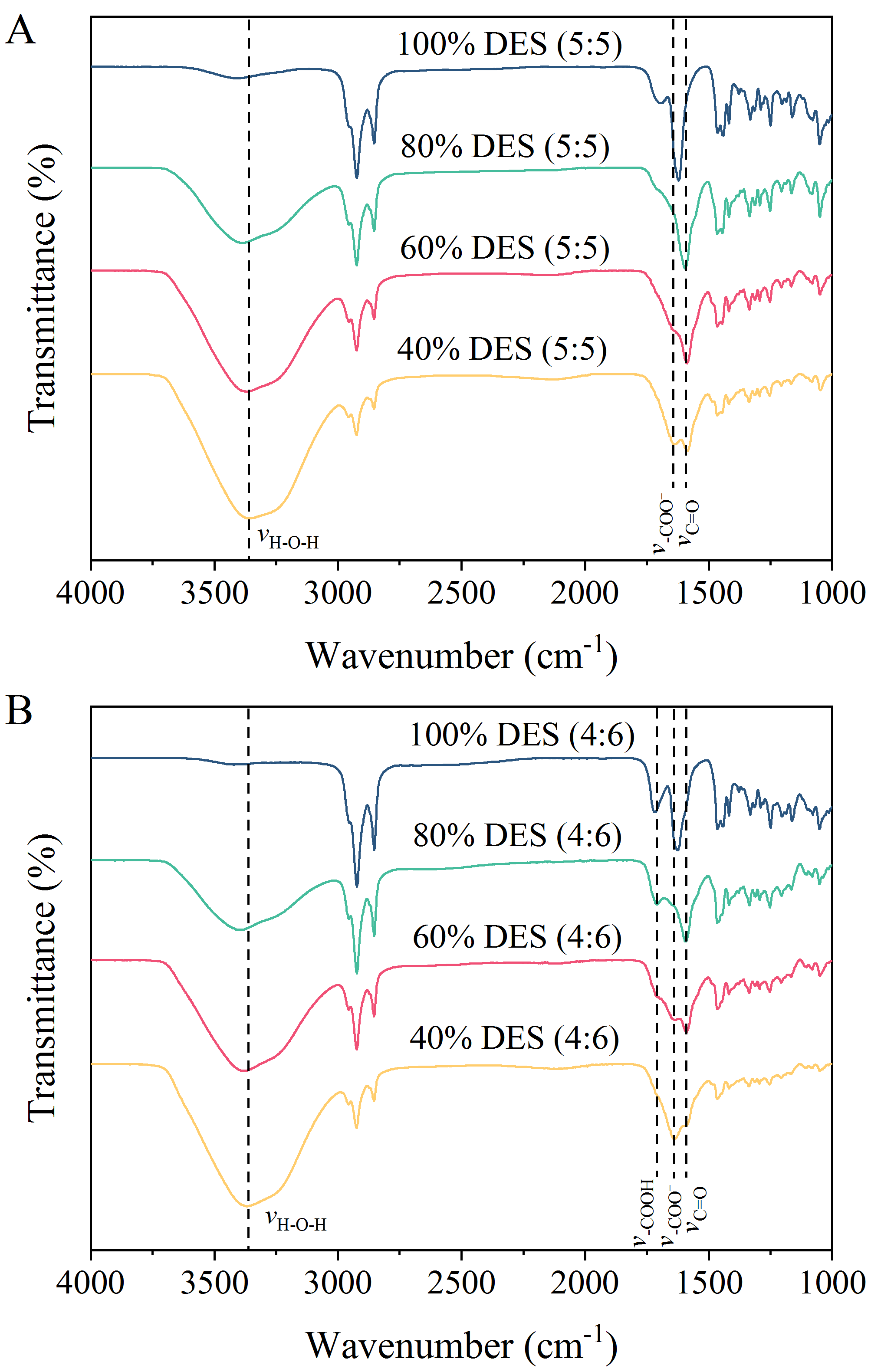


Figure S8. FTIR spectra for (A) DES (5:5) and (B) DES (4:6) at different water content. The stretching vibration intensity of LA carboxylate (-COO^−^) group increased with the increase in water content. The carbonyl (C=O) group of OMT and the hydroxyl groups (-OH) of water molecules shifted to lower wavenumbers, indicating that these functional groups participated in intermolecular hydrogen bonding with water molecules.
