# Supplementary material for: Deep eutectic solvent self-assembled reverse nanomicelles for transdermal delivery of sparingly soluble drugs
Source: J Nanobiotechnology. 2024 May 21;22:272. doi: 10.1186/s12951-024-02552-y (PMC11106993; doi:10.1186/s12951-024-02552-y)
Supplement: Supplementary file 6 — Supplementary Material 6 [file 12951_2024_2552_MOESM6_ESM.doc]

1. **The solubilization mechanism of DES-RM on TA**


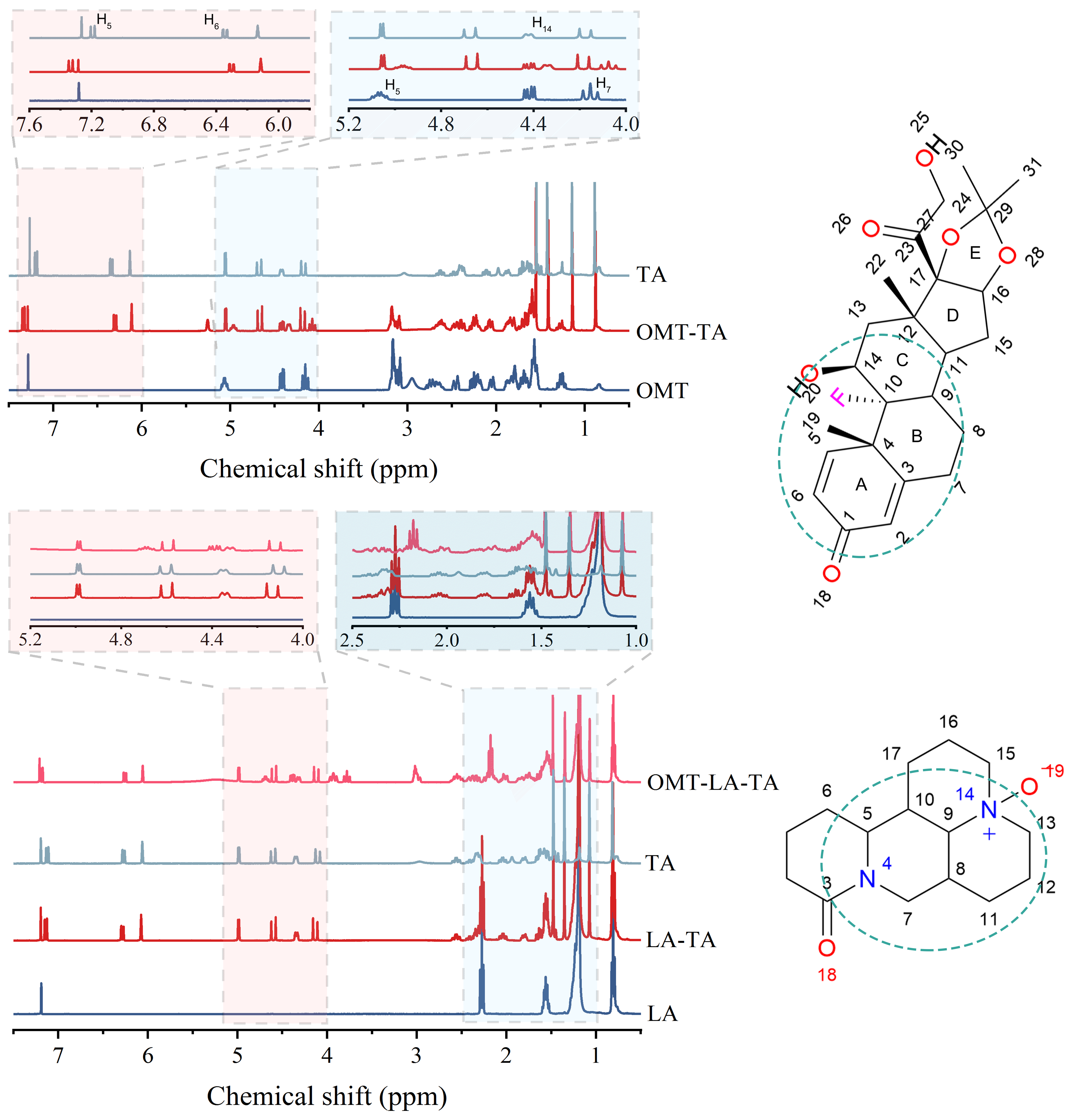
Figure S9. ^1^H NMR spectra (400 MHz, CDCl_3_) of TA recorded in the absence and presence of one equivalent of OMT molecule. The H_6_ and H_14_ proton signals of TA undergo a substantial upfield shift (Δ*δ* = -0.04 and -0.08 ppm) and the H_5_ proton signal exhibited a significant downfield shift (Δ*δ* = 0.15 ppm) when mixed with OMT. Such spectral differences, which possibly due to the intermolecular ring interaction and hydrogen bonding. Furthermore, the H_5_ and H_7_ proton peaks for OMT also shifted to upfield (Δ*δ* = -0.10 and -0.08 ppm), demonstrating a consequence of the π-π interaction between conjugated double-bond of TA and lactam structure of OMT. For comparison purposes, the interaction of TA with LA was also tested. Upon the addition of LA, no obvious changes (less than 0.01 ppm) in chemical shifts were observed for TA or LA. However, the changes in chemical shifts of TA were consistent with the trend in the OMT-TA mixture when OMT was added to the LA-TA mixture.

1. **The detailed attribution of the peaks observed in the ^1^H NMR spectrum**

OMT: ^1^H NMR (400 MHz, Chloroform-*d*) δ 5.07 (td, J = 5.5, 9.9 Hz, 1H, H_5_), 4.42 (dd, J = 5.2, 12.2 Hz, 1H, H_7_), 4.16 (t, J = 12.5 Hz, 12.2 Hz, 1H, H_7_), 3.05-3.22 (m, 4H, H_13_, H_15_), 2.95 (s, 1H), 2.62-2.81 (m, 2H), 2.45 (m, 1H), 2.23 (m, 2H), 2.06 (m, 1H), 1.59-1.93 (m, 6H), 1.54 (td, J = 3.0, 4.9, 5.7 Hz, 2H), 1.26 (tdd, J = 3.3, 9.7, 12.9 Hz, 1H), 0.85 (m, 1H) [1].

TA: ^1^H NMR (400 MHz, Chloroform-*d*) δ 7.19 (d, J = 10.1 Hz, 1H; H_5_), 6.34 (dd, J = 1.9, 10.1 Hz, 1H; H_6_), 6.13 (t, J = 1.8 Hz, 1H; H_2_), 5.06 (d, J = 5.0 Hz, 1H; H_16_), 4.68 (d, 1H, H_24_), 4.42 (d, J = 9.1 Hz, 1H; H_14_), 4.18 (d, 1H; H_24_), 3.04 (s, 1H), 2.63 (m, 1H; H_7_), 2.32-2.54 (m, 3H; H_7_, H_9_), 2.11 (td, J = 5.8, 12.7 Hz, 1H; H_13_), 1.98 (s, 1H; H_11_), 1.88 (dt, J = 5.3, 11.6 Hz, 1H; H_8_), 1.47-1.76 (m, 7H; H_8_, Me_19_, H_13_, H_15_), 1.43 (s, 3H; Me_31_), 1.15 (s, 3H; Me_30_), 0.89 (s, 3H; Me_22_) [2].

OMT-TA: ^1^H NMR (400 MHz, Chloroform-*d)* δ 7.34 (d, J = 10.1 Hz, 1H; H_5_), 6.30 (dd, J = 1.9, 10.1 Hz, 1H; H_6_), 6.12 (d, J = 1.7 Hz, 1H; H_2_), 5.25 (d, J = 3.6 Hz, 1H), 5.05 (d, J = 5.0 Hz, 1H; H_16_), 4.97 (td, J = 5.3, 9.9 Hz, 1H), 4.66 (d, 1H; H_24_), 4.42 (dd, J = 5.3, 12.3 Hz, 1H), 4.34 (m, 1H; H_14_), 4.18 (s, 1H; H_24_), 4.08 (t, J = 12.6 Hz, 1H), 3.06-3.23 (m, 5H), 2.63 (m, 3H), 2.33-2.52 (m, 3H), 2.24 (dtd, J = 4.2, 10.9, 11.4, 15.9 Hz, 3H), 2.08 (td, J = 5.5, 12.0, 12.6 Hz, 2H), 1.57-1.93 (m, 17H), 1.41 (s, 3H), 1.28 (m, 1H), 1.14 (s, 3H), 0.88 (s, 4H).

1. **MD simulation of TA-loaded DES-RM**

Firstly, the amorphous system consisting of OMT:LA:TA=40:60:3 molecules was generated at 298 K, followed by the energy minimization by the steepest descent and conjugate gradient methods. Therewith, NPT annealing dynamics was performed through 5 cycles between 300 and 500 K, with a total annealing time of 1000 ps. Finally, the optimized system was performed for 200 ps at the NPT ensemble run.

Next, TA@DES (4:6) was dispersed in an IPM box sized 7 × 7 × 7 nm, corresponding to the concentrations of 10% DES (4:6) and 0.5% TA. After minimization, the subsequent calculations were performed using the same procedure as the above simulation.

The results from Figure S10A showed that hydrogen bonds were formed between OMT and TA in DES. Moreover, there was also the dispersion interaction between the rings of OMT and TA. In Figure S10B, it can be seen that LA hydrophobic alkyl chain stretched towards the oil phase and OMT packed inside the core to form a reverse micelle-like structure, wherein TA could be adsorbed in the cores of RM or the interfacial layers of microdomains.


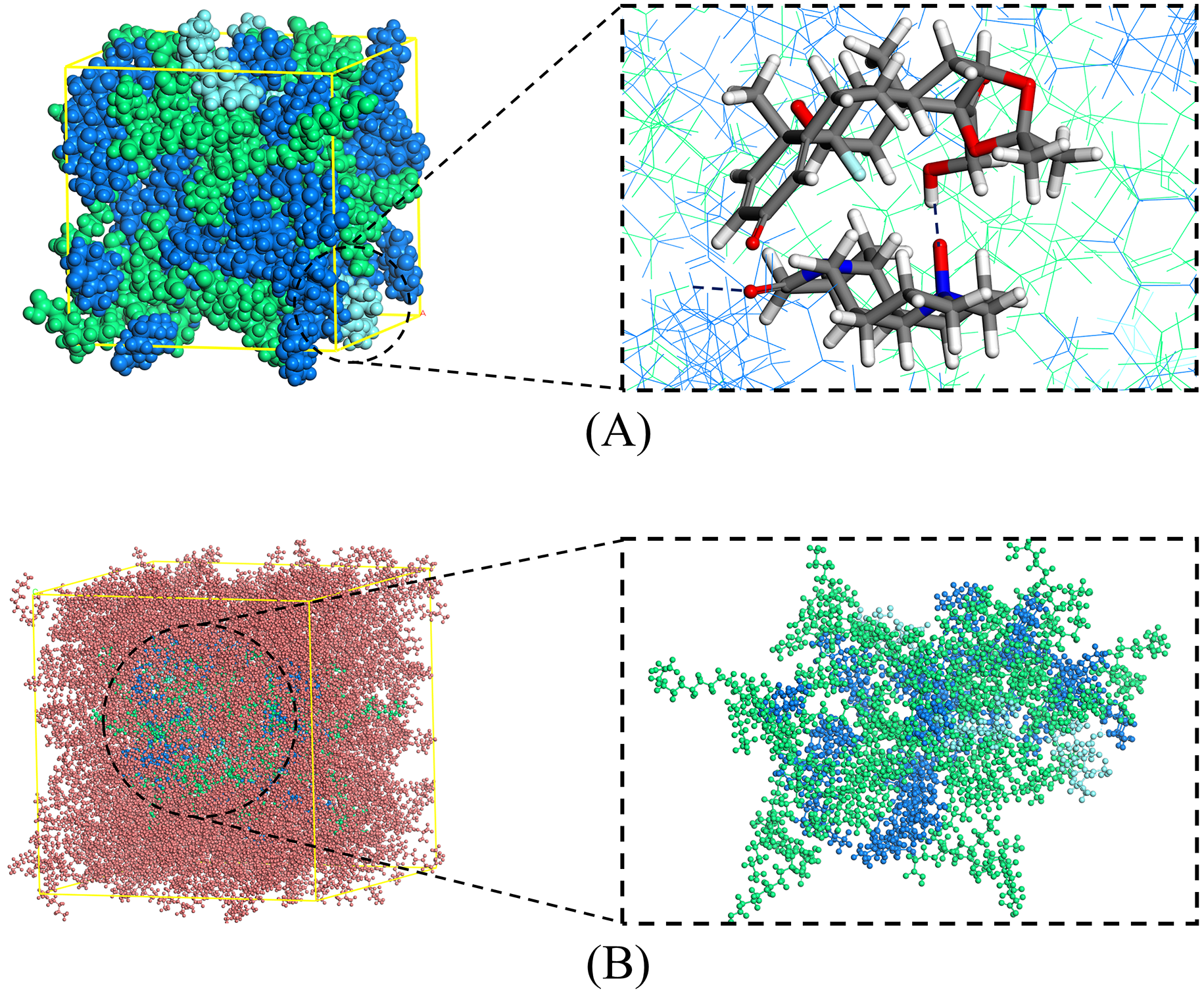


Figure S10. MD simulation of (A) TA-loaded DES (4:6) and (B) TA-loaded DES (4:6)-RM. Blue: OMT; green: LA; cyan: TA; brownish yellow: IPM.

**References**

1. D.Q. Huan, N.Q. Hop, N.T. Son, Oxymatrine: A current overview of its health benefits, Fitoterapia, 168 (2023).

2. A. Miro, F. Ungaro, F. Balzano, S. Masi, P. Musto, P. La Manna, G. Uccello-Barretta, F. Quaglia, Triamcinolone solubilization by (2-hydroxypropyl)-β-cyclodextrin: A spectroscopic and computational approach, Carbohydrate Polymers, 90 (2012) 1288-1298.
