# Supplementary material for: Deep eutectic solvent self-assembled reverse nanomicelles for transdermal delivery of sparingly soluble drugs
Source: J Nanobiotechnology. 2024 May 21;22:272. doi: 10.1186/s12951-024-02552-y (PMC11106993; doi:10.1186/s12951-024-02552-y)
Supplement: Supplementary file 7 — Supplementary Material 7 [file 12951_2024_2552_MOESM7_ESM.doc]

1. **Particle size and distribution of DES-RM and TA@DES-RM**





Figure S11. (A) Particle size and distribution of 10%, 30%, and 50% DES (4:6)-RM systems before (red) and after (blue) drug loaded. For 10% DES (4:6)-RM, the particle size increased significantly (*P* < 0.001) after TA loaded, which might be related to the adsorption of TA in the cores of RM or the interfacial layers of microdomains. For 30% DES (4:6)-RM, the presence of TA did not change much the particle size of RM much (*P* > 0.05), which might be due to higher TA solubility enhancing the miscibility and molecular interactions with DES. For 50% DES (4:6)-RM, the particle size also increased significantly (*P* < 0.05) after TA loaded, which is probably mainly caused by the transformation of the system structure to a bicontinuous phase. (B) Particle size and distribution of 10%, 30%, and 50% DES (4:6)-RM systems loaded with TA (0.1 wt%) before (red) and after (blue) freezing and thawing three times. (The column and line represent diameter and PDI).





Figure S12. TEM image of TA-loaded DES (4:6)-RM. The particle size was 9-16 nm.
