# Supplementary material for: Deep eutectic solvent self-assembled reverse nanomicelles for transdermal delivery of sparingly soluble drugs
Source: J Nanobiotechnology. 2024 May 21;22:272. doi: 10.1186/s12951-024-02552-y (PMC11106993; doi:10.1186/s12951-024-02552-y)
Supplement: Supplementary file 8 — Supplementary Material 8 [file 12951_2024_2552_MOESM8_ESM.doc]

1. **Skin penetration studies**


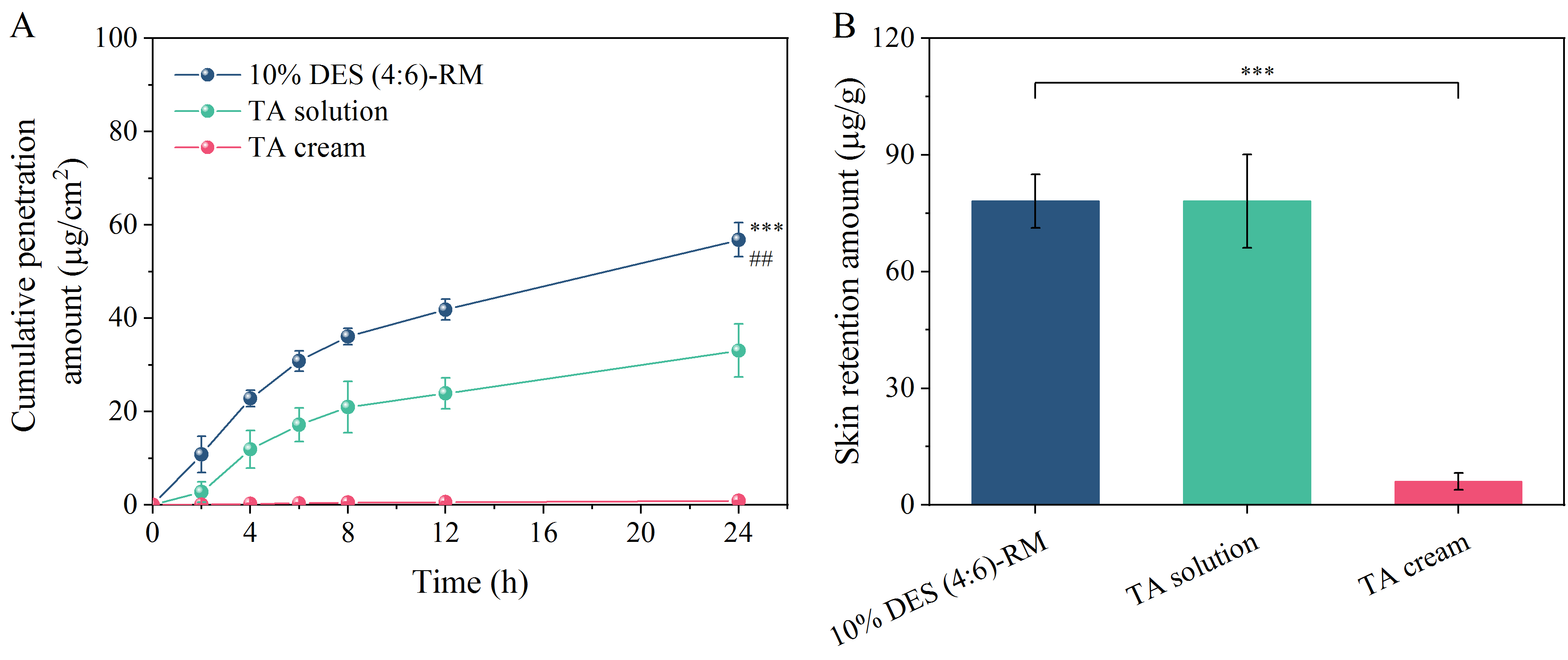


Figure S13. (A) TA cumulative penetration profiles and (B) skin retention form 10% DES (4:6)-RM compared to TA solution (HENGCHENG PHARMACEUTICAL, Guangdong, China) and TA cream (FRONT PHARMACEUTICAL, Anhui, China). ****P* < 0.001, compared to TA cream; ^##^*P* < 0.01, compared to TA solution.
