# Supplementary material for: Deep eutectic solvent self-assembled reverse nanomicelles for transdermal delivery of sparingly soluble drugs
Source: J Nanobiotechnology. 2024 May 21;22:272. doi: 10.1186/s12951-024-02552-y (PMC11106993; doi:10.1186/s12951-024-02552-y)
Supplement: Supplementary file 9 — Supplementary Material 9 [file 12951_2024_2552_MOESM9_ESM.doc]

1. **Skin penetration mechanism of DES-RM**


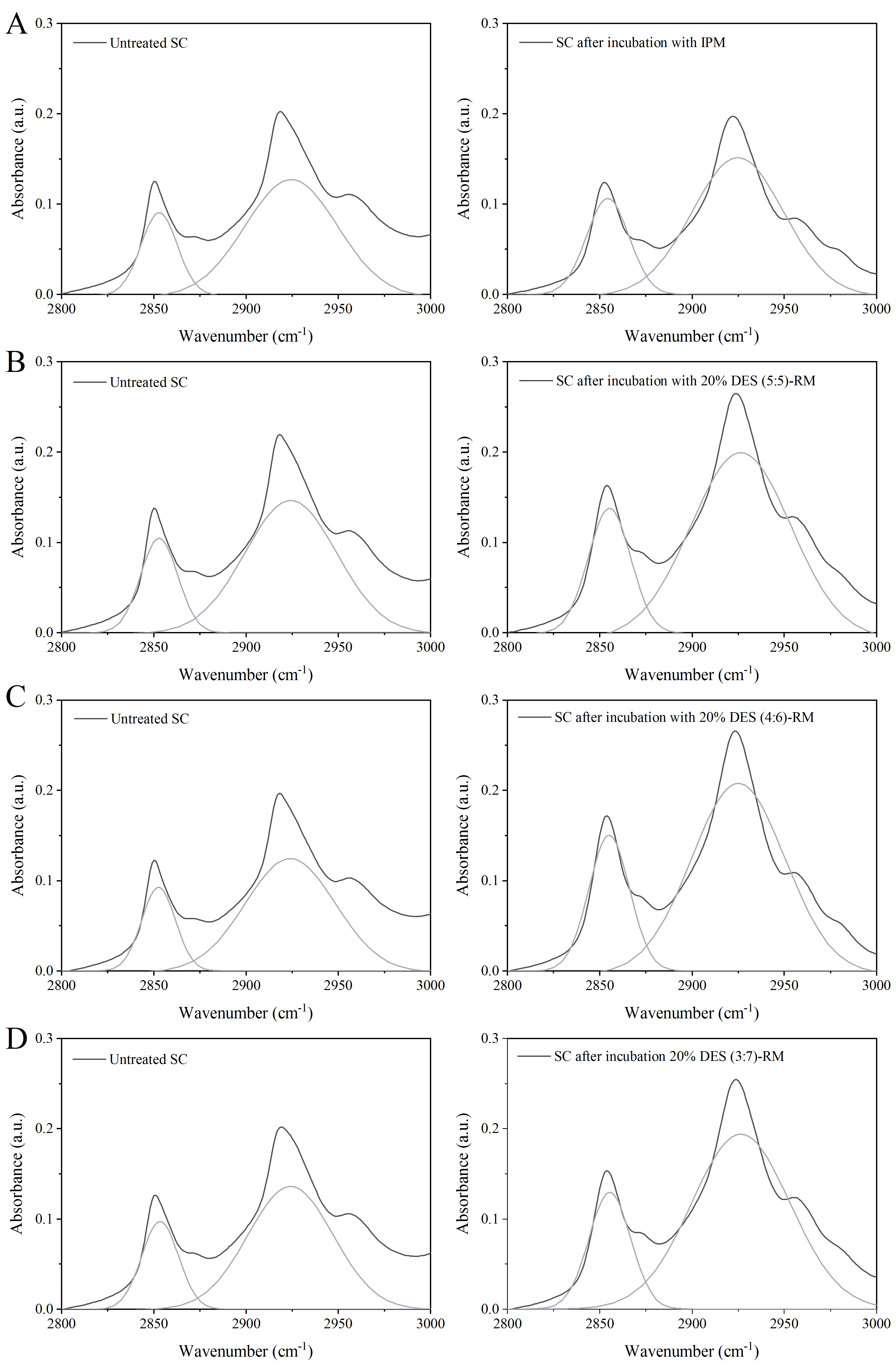


Figure S14. FTIR spectra for the CH_2_ symmetric and asymmetric stretching bands of SC lipids before and after incubation with (A) IPM, (B) DES (5:5)-RM, (C) DES (4:6)-RM, and (D) DES (3:7)-RM for 24 h. IPM or 20% DES-RM systems increased peak areas in the spectral region indicative of lipid content.
