# Supplementary material for: Deep eutectic solvent self-assembled reverse nanomicelles for transdermal delivery of sparingly soluble drugs
Source: J Nanobiotechnology. 2024 May 21;22:272. doi: 10.1186/s12951-024-02552-y (PMC11106993; doi:10.1186/s12951-024-02552-y)
Supplement: Supplementary file 10 — Supplementary Material 10 [file 12951_2024_2552_MOESM10_ESM.doc]

1. ***In vivo* safety assessment**

The biosafety of TA@DES-RM was evaluated by skin appearance, weight, blood biochemistry, and histological examination. The BALB/c mice were randomly divided into four groups (n = 3). The mice that used normal saline served as the control group. The mice in the other three groups were administered different TA formulations: (1) control group; (2) TA commercial solution group (namely CS, 50 mg); (3) low-dose TA@DES-RM group (50 mg); and (4) high-dose TA@DES-RM group (100 mg). The body weights of the mice were recorded daily using a weighing scale. Blood samples of different groups were collected via retro-orbital bleeding after 5 days. Then, the serum was acquired by centrifugation for 15 min at 3000 rpm and 4 °C. Creatinine, urea nitrogen, aspartate aminotransferase, and alanine aminotransferase were assayed using a fully automatic biochemistry analyzer. After the mice were sacrificed, the back skin and major organs of the mice were collected and fixed with 4% paraformaldehyde for 24 h, embedded in paraffin, and then sliced for H&E staining and further observation.

The skin appearance, weight, and blood biochemical indicators of the mice in low-dose and high-dose groups showed no obvious difference compared to the control group during the experimental period. Moreover, H&E staining of major organs of the mice did not show obvious tissue injuries, demonstrating the outstanding biocompatibility of the oil-based DES system. However, skin from TA CS-treated mice presented significant edema, epidermal thickening, as well as elevated CREA levels, indicating that there was skin irritation and slight renal toxicity. This may be attributed to the presence of a high concentration of ethanol in TA CS.


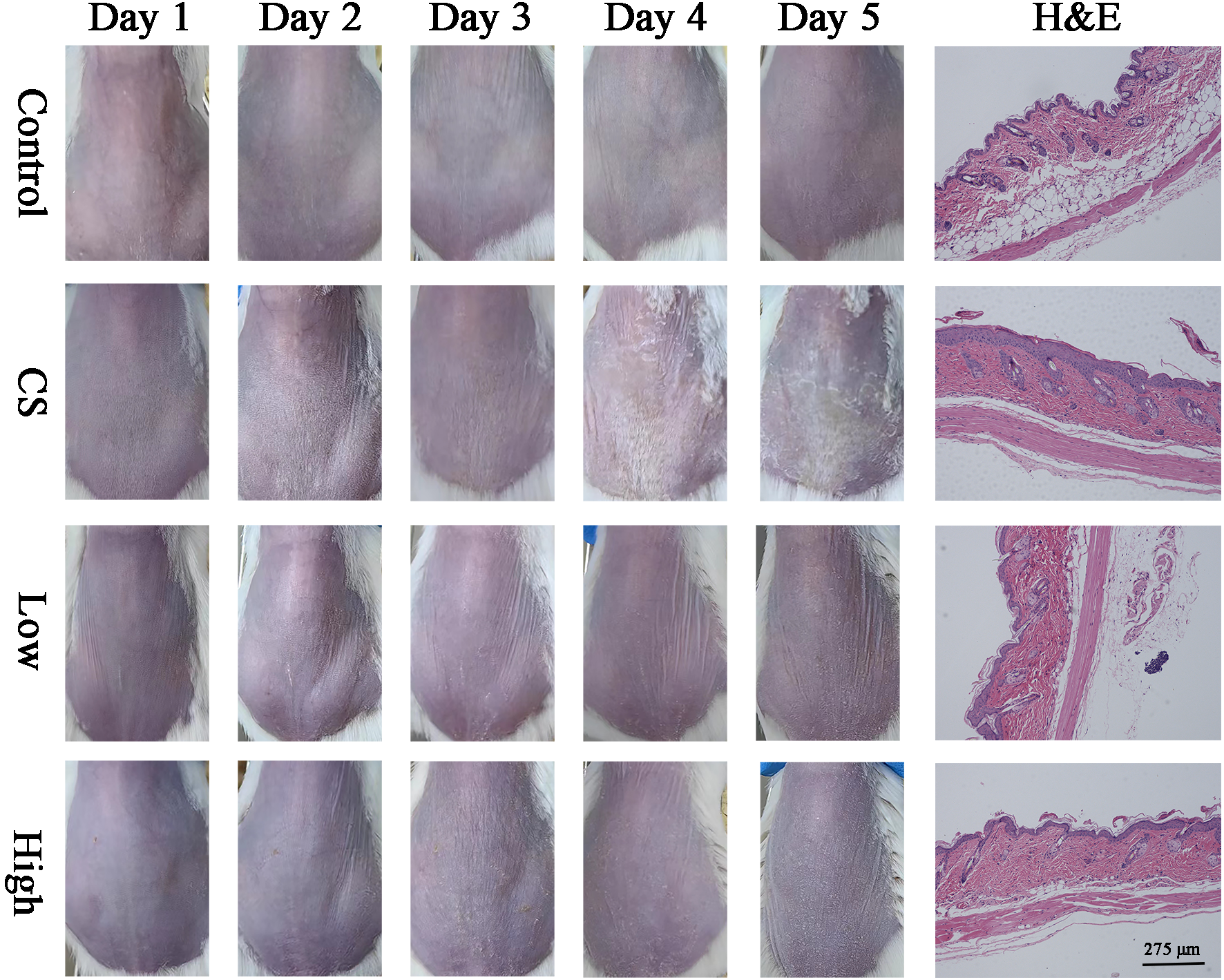


Figure S15. The macroscopic appearance and H&E staining histological appearance of after 5 days of the mice skin. Scale bar set at 275 μm for magnification at 10x.





Figure S16. Body weight and blood biochemical indicators of the mice. Abbreviations: CREA, creatinine; BUN, urea nitrogen; AST, aspartate aminotransferase; ALT, alanine aminotransferase. ns, not statistically significant; ***P* < 0.01, compared to the control group.


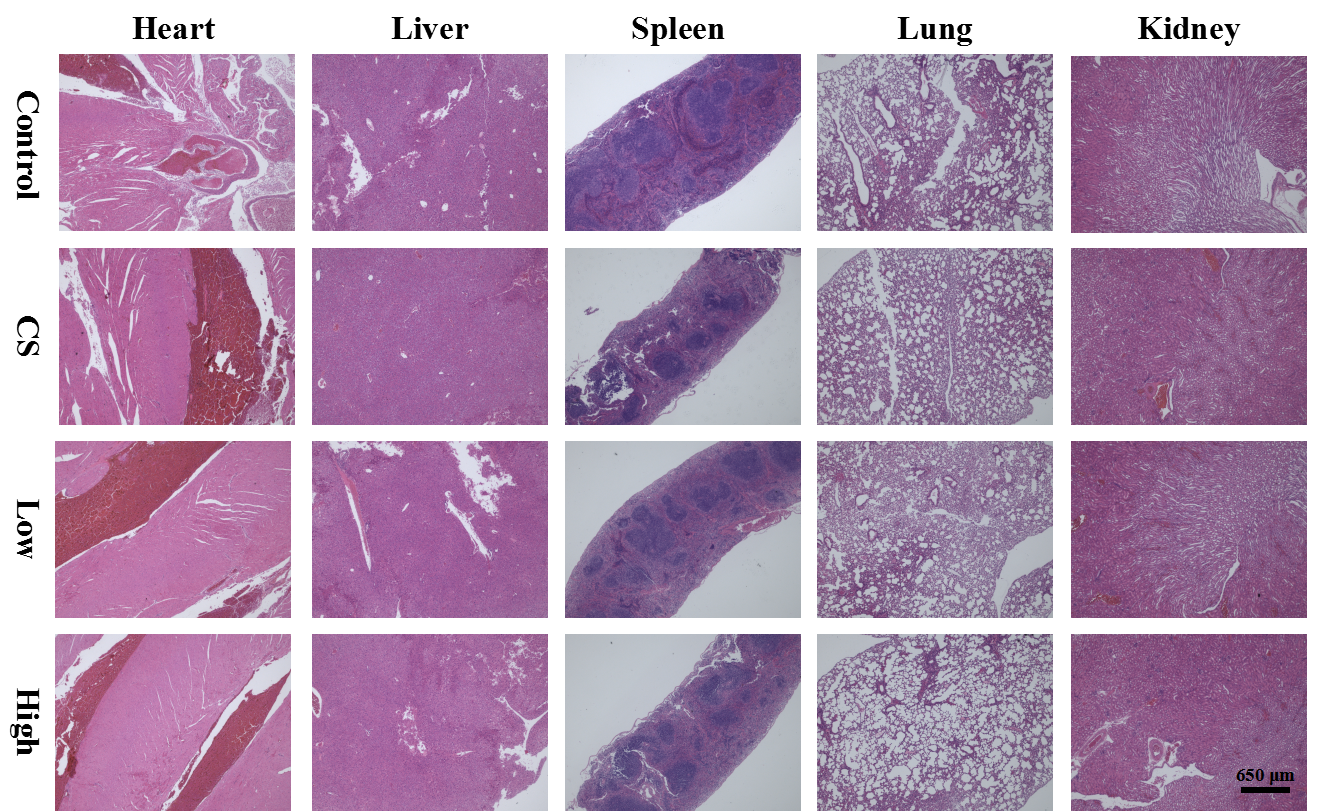


Figure S17. H&E staining of major organs (i.e., heart, liver, spleen, lung, and kidney) of the mice. Scale bar set at 650 μm for magnification at 4x.
